# Cfs1p, a Novel Membrane Protein in the PQ-Loop Family, Is Involved in Phospholipid Flippase Functions in Yeast

**DOI:** 10.1534/g3.116.035238

**Published:** 2016-11-08

**Authors:** Takaharu Yamamoto, Konomi Fujimura-Kamada, Eno Shioji, Risa Suzuki, Kazuma Tanaka

**Affiliations:** * Graduate School of Life Science, Division of Molecular Interaction, Institute for Genetic Medicine, Hokkaido University, Sapporo 060-0815, Japan; † Graduate School of Medicine, Division of Molecular Interaction, Institute for Genetic Medicine, Hokkaido University, Sapporo 060-0815, Japan

**Keywords:** phospholipid asymmetry, P4-ATPase, membrane transport, floppase, scramblase

## Abstract

Type 4 P-type ATPases (P4-ATPases) function as phospholipid flippases, which translocate phospholipids from the exoplasmic leaflet to the cytoplasmic leaflet of the lipid bilayer, to generate and maintain asymmetric distribution of phospholipids at the plasma membrane and endosomal/Golgi membranes. The budding yeast *Saccharomyces cerevisiae* has four heteromeric flippases (Drs2p, Dnf1p, Dnf2p, and Dnf3p), associated with the Cdc50p family noncatalytic subunit, and one monomeric flippase, Neo1p. They have been suggested to function in vesicle formation in membrane trafficking pathways, but details of their mechanisms remain to be clarified. Here, to search for novel factors that functionally interact with flippases, we screened transposon insertional mutants for strains that suppressed the cold-sensitive growth defect in the *cdc50*Δ mutant. We identified a mutation of *YMR010W* encoding a novel conserved membrane protein that belongs to the PQ-loop family including the cystine transporter cystinosin and the SWEET sugar transporters. We named this gene *CFS1* (*cdc fifty* suppressor 1). GFP-tagged Cfs1p was partially colocalized with Drs2p and Neo1p to endosomal/late Golgi membranes. Interestingly, the *cfs1*Δ mutation suppressed growth defects in all flippase mutants. Accordingly, defects in membrane trafficking in the flippase mutants were also suppressed. These results suggest that Cfs1p and flippases function antagonistically in membrane trafficking pathways. A growth assay to assess sensitivity to duramycin, a phosphatidylethanolamine (PE)-binding peptide, suggested that the *cfs1*Δ mutation changed PE asymmetry in the plasma membrane. Cfs1p may thus be a novel regulator of phospholipid asymmetry.

In eukaryotic cells, phospholipids are asymmetrically distributed across the cell-membrane bilayer. Phosphatidylserine (PS) and phosphatidylethanolamine (PE) are enriched in the cytoplasmic leaflet of the plasma membrane, whereas phosphatidylcholine (PC) and sphingolipids are located in the exoplasmic leaflet (Op den Kamp 1979). Because of the amphipathic nature of phospholipids, their transverse movement is catalyzed by three classes of proteins: flippases, floppases, and scramblases (Daleke 2003). Scramblases are energy-independent and bidirectional transporters that dissipate phospholipid asymmetry. PS exposure induced by scramblases is necessary for functions such as blood coagulation and the recognition of apoptotic or aged cells (Segawa and Nagata 2015). Flippases and floppases are energy-dependent transporters that catalyze inward (exoplasmic to cytoplasmic) and outward movement of phospholipids, respectively, and are involved in the establishment and maintenance of phospholipid asymmetry at the plasma membrane and intracellular organelle membranes (Coleman *et al.* 2013; Hankins *et al.* 2015). Floppase activities are catalyzed by ATP-binding cassette (ABC) transporters, some of which also catalyze flippase activities (López-Marqués *et al.* 2015).

P4-ATPases are phospholipid flippases. In mammals, they have been suggested to be involved in intrahepatic cholestasis (Bull *et al.* 1998; Klomp *et al.* 2004), diabetes (Dhar *et al.* 2004), B cell development (Siggs *et al.* 2011; Yabas *et al.* 2011), and axonal degeneration (Zhu *et al.* 2012) (reviewed in van der Mark *et al.* 2013), but the molecular mechanisms that underlie these cellular functions remain to be elucidated. The yeast *Saccharomyces cerevisiae* encodes five P4-ATPases: Drs2p, Dnf1p, Dnf2p, Dnf3p, and Neo1p (Tanaka *et al.* 2011). Of these, Drs2p, Dnf1p/Dnf2p, and Dnf3p form complexes with noncatalytic subunits of the Cdc50p family: Cdc50p, Lem3p, and Crf1p, respectively. These interactions are required for ER exit, proper localization, function, and activity of the phospholipid flippases (Saito *et al.* 2004; Noji *et al.* 2006; Furuta *et al.* 2007; Lenoir *et al.* 2009; Takahashi *et al.* 2011; Puts *et al.* 2012). Therefore, *drs2*Δ, *dnf1*Δ *dnf2*Δ, and *dnf3*Δ mutants are phenocopied by *cdc50*Δ, *lem3*Δ, and *crf1*Δ mutants, respectively (Saito *et al.* 2004; Furuta *et al.* 2007).

Phenotypic analyses of yeast phospholipid flippase mutants suggest that they function in membrane trafficking pathways (Tanaka *et al.* 2011; Sebastian *et al.* 2012). Cdc50p-Drs2p, Lem3p-Dnf1p/Dnf2p, and Crf1p-Dnf3p are collectively essential for viability and are required for retrieval from early endosomes to the *trans*-Golgi network (TGN) during the endocytic recycling pathway (Furuta *et al.* 2007). Cdc50p-Drs2p plays a prominent role in this pathway and is also involved in the formation of clathrin-coated vesicles from early endosomal/TGN membranes (Chen *et al.* 1999; Gall *et al.* 2002), but the underlying mechanisms are unknown.

Neo1p does not associate with a Cdc50p family member (Saito *et al.* 2004; Furuta *et al.* 2007) and is independently essential for viability. Neo1p is involved in membrane trafficking from the *cis*-Golgi to the ER and within the endosomal/Golgi system (Hua and Graham 2003; Wicky *et al.* 2004). Although the phospholipid flipping activity of Neo1p has not been demonstrated, Neo1p functions redundantly with Cdc50p-Drs2p in the endocytic recycling pathway (Takeda *et al.* 2014).

To further understand the functions of flippases and regulatory mechanisms of phospholipid asymmetry, it is important to identify novel machinery functionally associated with flippases. In this study, we performed a screen for suppressor mutations of a cold-sensitive growth defect in the *cdc50*Δ mutant. This resulted in identification of a mutation in an uncharacterized gene, *YMR010W*, encoding a novel membrane protein of the PQ-loop family. Our genetic analyses revealed that Ymr010wp functions antagonistically to phospholipid flippases.

## Materials and Methods

### Genetic methods and growth assay

Chemicals were purchased from Wako Pure Chemicals Industries (Osaka, Japan) unless otherwise described. Duramycin was purchased from Sigma-Aldrich (St. Louis, MO). Yeast strains were cultured in rich YPDA [1% yeast extract (Difco Laboratories, Detroit, MI), 2% bacto-peptone (Difco), 2% glucose, and 0.01% adenine] or YPGA (1% yeast extract, 2% bacto-peptone, 3% galactose, 0.2% sucrose, and 0.01% adenine) medium. When a tryptophan requirement was examined, YPDA was additionally supplemented with 200 μg/ml tryptophan (YPDAW). Standard genetic manipulations and plasmid transformation of yeast were performed as described previously (Elble 1992; Guthrie and Fink 2002). Synthetic glucose (SD) medium containing the required nutrient (Guthrie and Fink 2002) was used for a genetic screen and fluorescent microscopy. To assay growth of *P_GAL1_-3HA-CDC50* strains carrying *TRP1*-harboring or *URA3*-harboring plasmids, yeast transformants were selected on synthetic SGA-Trp [0.67% yeast nitrogen base w/o amino acids (Difco), 0.5% casamino acids (Difco), 3% galactose, 0.2% sucrose, 0.03% uracil, and 0.01% adenine] or SGA-Ura (0.67% yeast nitrogen base w/o amino acids, 0.5% casamino acids, 3% galactose, 0.2% sucrose, 0.03% tryptophan, and 0.01% adenine) medium, respectively, and then examined for growth on SDA-Trp (0.67% yeast nitrogen base w/o amino acids, 0.5% casamino acids, 2% glucose, 0.03% uracil, and 0.01% adenine) or SDA-Ura (0.67% yeast nitrogen base w/o amino acids, 0.5% casamino acids, 2% glucose, 0.03% tryptophan, and 0.01% adenine) medium, respectively. For serial dilution spot assay, cells were grown to early log phase in appropriate medium, washed with YP (1% yeast extract and 2% bacto-peptone), and adjusted to a concentration of 0.1 OD_600_/ml. From fivefold dilutions, 4 μl drops were spotted onto appropriate plates, followed by incubation under the indicated conditions.

### Yeast strains and plasmids

Yeast strains used in this study are listed in Supplemental Material, Table S1. Standard molecular biological techniques (Sambrook and Russell 2001) were used for the construction of plasmids, PCR amplification, and DNA sequencing. Gene deletions of *CFS1*, *KES1*, *FUN26*, and *PLB3* in the YEF473 (Bi and Pringle 1996) genetic background were performed as follows. The regions containing the *KanMX4* disruption marker and the flanking sequences were PCR-amplified using genomic DNA derived from the knockout strain in the BY4741 (Brachmann *et al.* 1998) strain background (a gift from Charles Boone, University of Toronto) as a template. The amplified DNA fragments were introduced into the appropriate strains, and G418-resistant transformants were selected. Yeast strains carrying C-terminally green fluorescent protein (GFP)-tagged *ENA1*, C-terminally enhanced GFP (EGFP)-tagged *CFS1*, and C-terminally monomeric red fluorescent protein 1 (mRFP1)-tagged genes (*DRS2*, *NEO1*, and *SEC7*) were constructed by the PCR-based procedure as previously described (Longtine *et al.* 1998). All strains constructed by the PCR-based procedure were verified by colony PCR amplification to confirm that replacement or insertion had occurred at the expected loci. The *sec14-3* mutant in the YEF473 genetic background was constructed by backcrossing the original mutant (a gift from Randy Schekman) to our wild-type strain (YKT1066) three times. The GFP-tagged Lact-C2 plasmid (pRS416-P_GPD_-GFP-Lact-C2) (Yeung *et al.* 2008) was purchased from Hematologic Technologies, Inc. (Essex Junction, VT). *CFS1* and *KES1* genes were amplified by PCR, and subcloned into a centromeric plasmid pRS314 (Sikorski and Hieter 1989) using appropriate restriction enzymes to construct pRS314-CFS1 and pRS314-KES1, which were sequenced to confirm that no mutation had occurred in the PCR process. Each gene fragment was also subcloned to a multicopy plasmid, YEplac195 (Gietz and Sugino 1988), to construct YEplac195-CFS1 and YEplac195-KES1.

### Screening for mutants that overcome defects by the cdc50Δ mutation

**Figure 1:**
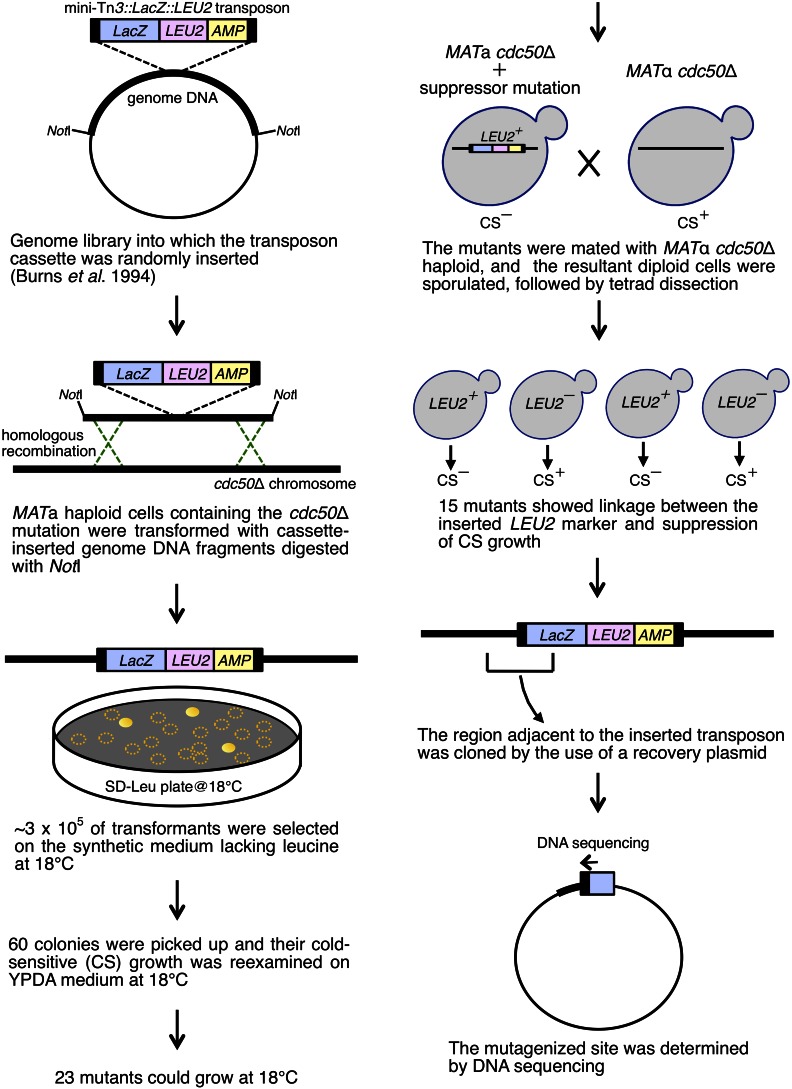
Overall scheme of the screen for mutations that suppress the CS growth defect in the *cdc50*Δ mutant. CS, cold-sensitive; YPDA, yeast extract peptone glucose adenine medium.

Screening for mutations that suppress the cold-sensitive growth defect in the *cdc50*Δ mutant was performed using a genomic library (kindly provided by Michael Snyder, Stanford University) that had been mutagenized by random insertion of the mini-Tn*3*::*LacZ*::*LEU2* transposon cassette (Burns *et al.* 1994). The overall scheme of the screen is shown in Figure 1. Twenty-four micrograms of the genomic library was digested with *Not*I, and 6 × 10^9^ cells of YKT249 were transformed with the resulting DNA fragments by the high efficiency transformation protocol (Gietz *et al.* 1995). Approximately 3 × 10^5^ of transformants were spread onto SD-Leu plates, followed by incubation at 18° for 4 d. Of 60 mutants that formed colonies, 15 mutants grew well at 18° after restreaking on YPDA plates, and showed linkage between the inserted *LEU2* marker and suppression of the cold-sensitive growth defect by tetrad-analysis. To determine the mutagenized locus, the genomic DNA adjacent to the inserted transposon was cloned into a recovery plasmid (a gift from Akio Kihara, Hokkaido University) from each mutant, followed by sequence analyses.

### Microscopic observations

Cells expressing fluorescent proteins were observed using a Nikon ECLIPSE E800 microscope equipped with a 1.4 numerical aperture 100 × Plan Apo oil immersion objective lens with appropriate fluorescence filter sets or differential interference contrast (DIC) optics (Nikon Instec, Tokyo, Japan). Images were acquired using a cooled digital charge-coupled device camera (C4742-95-12NR; Hamamatsu Photonics, Hamamatsu, Japan) and AQUACOSMOS software (Hamamatsu Photonics) with 1 × 1 binning.

GFP-Snc1p, GFP-Lact-C2, and Ena1p-GFP were observed in living cells, which were grown as described in figure legends, harvested, and resuspended in SD medium. Cells were immediately observed using a GFP bandpass filter set. Colocalization of Cfs1p-EGFP with Drs2p-mRFP1, Neo1p-mRFP1, or Sec7p-mRFP1 was examined in fixed cells. Fixation was performed for 10 min at 25° by direct addition of 37% formaldehyde to a final concentration of 0.2% (Drs2p-mRFP1 and Neo1p-mRFP1) or 2% (Sec7p-mRFP1) in the culture medium. After fixation, cells were washed with phosphate-buffered saline and immediately observed using a GFP bandpass or a G2-A (for mRFP1) filter set.

### Data availability

Strains and plasmids are available upon request. Table S1 contains genotypes and resources or references for each yeast strain used in this study. The authors state that all data necessary for confirming the conclusions presented in the article are represented fully within the article and supplemental files including Figure S1, Figure S2, Figure S3, Figure S4, Figure S5, and Figure S6.

## Results

### Identification of mutations that suppress the cold-sensitive growth defect in the cdc50Δ mutant

**Figure 2:**
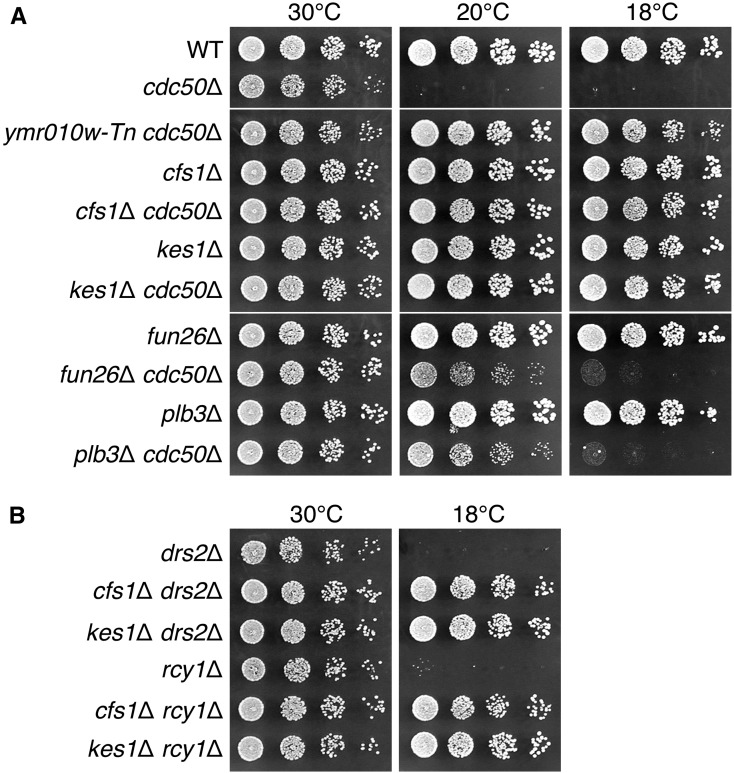
Identification of mutations that suppress the cold-sensitive growth defect in the *cdc50*Δ mutant. (A) Suppression of the cold-sensitive growth defect in the *cdc50*Δ mutant by complete gene disruption of the identified genes. Fivefold serial dilutions of exponentially growing cultures were spotted onto YPDA plates, followed by incubation at 30° for 1.5 d, or at 20 or 18° for 5 d. The strains used were WT (YKT1066), *cdc50*Δ (YKT1507), *ymr010w-Tn cdc50*Δ (YKT2024), *cfs1*Δ (YKT2070), *cfs1*Δ *cdc50*Δ (YKT2025), *kes1*Δ (YKT2035), *kes1*Δ *cdc50*Δ (YKT2026), *fun26*Δ (YKT2029), *fun26*Δ *cdc50*Δ (YKT2030), *plb3*Δ (YKT2031), and *plb3*Δ *cdc50*Δ (YKT2032). These strains were in the *TRP1* background, because the *kes1*Δ mutant containing the *trp1*Δ mutation requires additional supplementation of tryptophan for growth on standard rich medium (Jiang *et al.* 1994). (B) The *cfs1*Δ mutation suppresses cold-sensitive growth defects in the *drs2*Δ and *rcy1*Δ mutants. Cell growth was examined as in (A). The strains used, all of which were in the *TRP1* background, were *drs2*Δ (YKT1636), *cfs1*Δ *drs2*Δ (YKT2081), *kes1*Δ *drs2*Δ (YKT2082), *rcy1*Δ (YKT2039), *cfs1*Δ *rcy1*Δ (YKT2083), and *kes1*Δ *rcy1*Δ (YKT2084). WT, wild-type; YPDA, yeast extract peptone glucose adenine medium.

The disruption of the *CDC50* gene, which encodes a noncatalytic subunit of the Drs2p phospholipid flippase catalytic subunit, leads to a cold-sensitive growth defect (Misu *et al.* 2003; Saito *et al.* 2004). To search for genes with phospholipid flippase-related functions, we performed a screen for mutations that suppress the cold-sensitive growth defect in the *cdc50*Δ mutant by using transposon mutagenesis as described in *Materials and Methods* (Figure 1). As shown in Table 1, 15 isolated mutations were divided into seven classes. To examine whether complete gene disruption of the identified gene can suppress the cold-sensitive growth defect, a complete disruptant of each gene was constructed and crossed to the *cdc50*Δ mutant. After isolation of double mutants by tetrad dissection, their growth was examined. The *ymr010w*Δ mutation strongly suppressed the cold-sensitive growth defect as the original *ymr010w-Tn* mutation isolated in the screening (Figure 2A). We named *YMR010W* *CFS1*, which stands for Cdc Fifty (*cdc50*) Suppressor 1. The *kes1*Δ mutation also strongly suppressed *cdc50*Δ as reported previously for its suppression of *drs2*Δ (Muthusamy *et al.* 2009), whereas the *fun26*Δ or *plb3*Δ mutation weakly suppressed it (Figure 2A). The *alg6*Δ and *hmg1*Δ mutations did not suppress it (data not shown). The *rix1*Δ mutation was not examined.

**Table 1:** Identified mutations that suppress the cold-sensitive growth defect in the *cdc50*Δ mutant

Standard, Alias, or Systematic Name	Number of Isolated Insertional Mutation	Functional Description
*YMR010W* (*CFS1*)	1	Member of the PQ-loop family
*KES1* (*OSH4*)	1	Oxysterol-binding protein (OSBP) homolog (Jiang *et al.* 1994; Beh *et al.* 2001)
*FUN26*	4	Nucleoside and nucleobase transporter (Vickers *et al.* 2000), and nicotinamide riboside transporter (Lu and Lin 2011)
*PLB3*	4	Phospholipase B (Merkel *et al.* 1999)
*ALG6*	2	α-1,3-glucosyltransferase
*HMG1*	2	HMG-CoA reductase, which functions in a rate-limiting step of ergosterol biosynthesis
*RIX1*	1	Component of the Rix1 complex required for the processing of 35S pre-rRNA (ribosomal RNA) in pre-60S ribosomal particles and for the initiation of DNA replication

**Figure 3:**
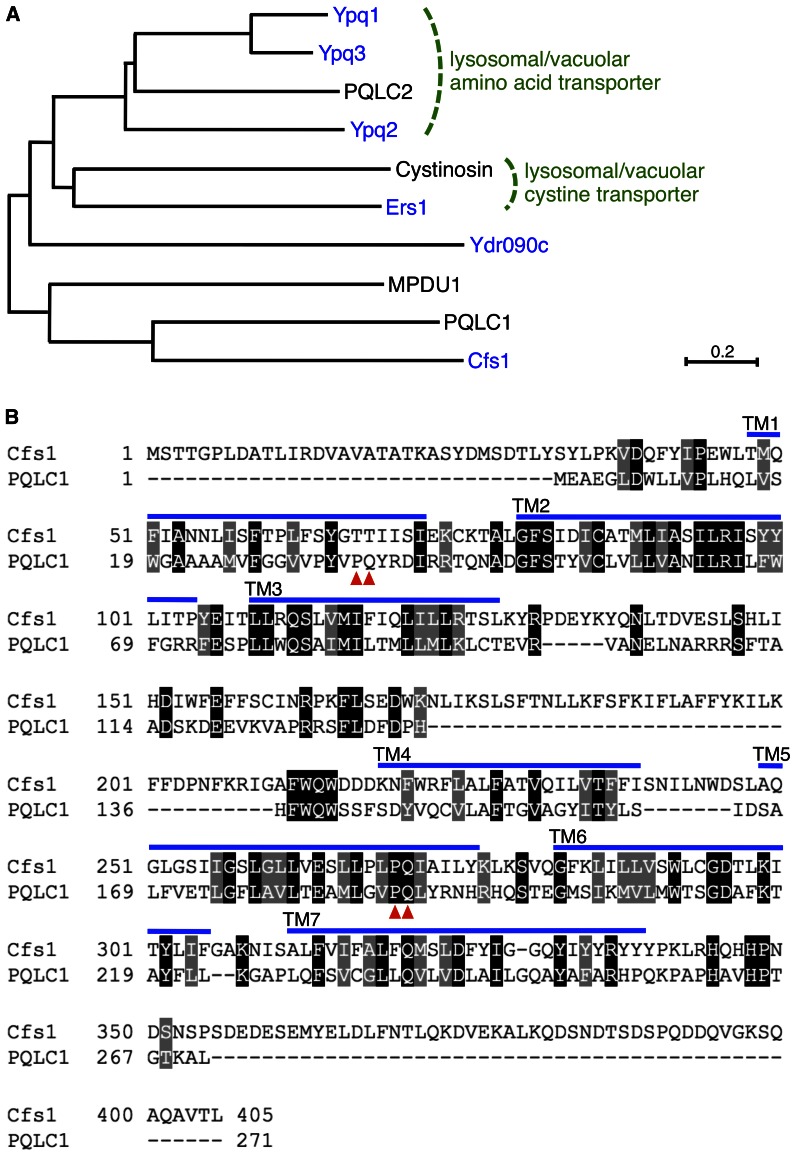
Cfs1p is a member of the PQ-loop protein family. (A) Phylogenetic tree of yeast PQ-loop proteins and representatives of human homologs. It was constructed by the neighbor-joining method (Saitou and Nei 1987) using the MEGA7 software (http://www.megasoftware.net/), and branch lengths reflect the estimated amino acid substitutions per site (see scale bar). NCBI (National Center for Biotechnology Information) accession versions of the proteins are: *Homo sapiens* (black): PQLC1 (NP_079354.2), PQLC2 (Q6ZP29.1), Cystinosin (CAA11021.1), and MPDU1 (NP_004861.1); *S. cerevisiae* (blue): Ypq1 (KZV07787.1), Ypq2 (KZV12591.1), Ypq3 (P38279.1), Ers1 (KZV12920.1), Ydr090c (AAS56014.1), and Cfs1 (Ymr010w, AAS56443.1). (B) Comparison of the amino acid sequences of Cfs1p and its nearest human protein PQLC1. Full-length amino acid sequences were initially aligned using the CLUSTAL W program (http://www.clustal.org/) and the alignment was optimized by the BOXSHADE program (http://embnet.vital-it.ch/software/BOX_form.html). Black and gray boxes indicate identical and similar amino acids, respectively. Transmembrane regions were predicted using the Philius transmembrane prediction server (http://www.yeastrc.org/philius/pages/philius/runPhilius.jsp) and modified by referring to a previous study (Saudek 2012). Blue lines and red arrowheads indicate predicted transmembrane regions and the PQ-motif conserved among the PQ-loop protein family, respectively.

**Figure 4:**
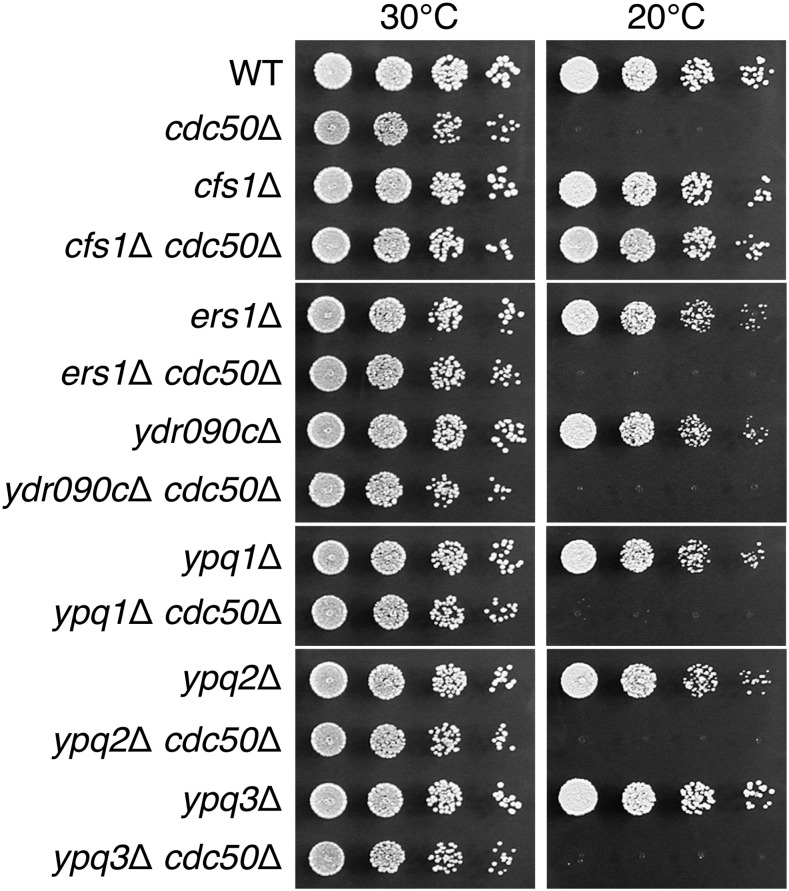
Only the *cfs1*Δ mutation suppresses the growth defect of the *cdc50*Δ mutant among PQ-loop family members. Fivefold serial dilutions of exponentially growing cultures were spotted onto YPDA plates, followed by incubation at 30° for 1.5 d or at 20° for 5 d. The strains used were WT (KKT3), *cdc50*Δ (KKT9), *cfs1*Δ (KKT479), *cfs1*Δ *cdc50*Δ (KKT480), *ers1*Δ (KKT481), *ers1*Δ *cdc50*Δ (KKT482), *ydr090c*Δ (KKT483), *ydr090c*Δ *cdc50*Δ (KKT484), *ypq1*Δ (KKT485), *ypq1*Δ *cdc50*Δ (KKT486), *ypq2*Δ (KKT487), *ypq2*Δ *cdc50*Δ (KKT488), *ypq3*Δ (KKT489), and *ypq3*Δ *cdc50*Δ (KKT490). WT, wild-type; YPDA, yeast extract peptone glucose adenine medium.

In this study, we focused our analysis on functions of *CFS1*. Cfs1p is a member of the “PQ-loop family,” which has a seven-helix membrane topology and is characterized by the presence of a duplicated motif termed the “PQ-loop” (Jézégou *et al.* 2012) (Figure 3). Budding yeast has six PQ-loop proteins (Figure 3A). Ers1p transports cystine (Simpkins *et al.* 2016), and is a functional homolog of mammalian cystinosin, mutations in which causes the lysosomal storage disorder cystinosis (Gao *et al.* 2005). Ypq1p/Ypq2p/Ypq3p and their mammalian homolog PQLC2 are vacuolar/lysosomal cationic amino acid exporters (Jézégou *et al.* 2012). Ypq1p/Ypq2p/Ypq3p are also implicated in uptake of basic amino acids in the vacuolar membrane vesicles (Sekito *et al.* 2014; Manabe *et al.* 2016). In contrast to these PQ-loop proteins, the activities of Cfs1p and its nearest human protein PQLC1 remain to be clarified. Cfs1p is unique in that it lacks the N-terminal PQ-loop motif (Figure 3B). Disruption of these *CFS1* homologs did not suppress the cold-sensitive growth defects in the *cdc50*Δ mutant (Figure 4), suggesting that the phospholipid flippase-related function is unique to Cfs1p among the PQ-loop family members.

The *cfs1*Δ mutation, as well as the *kes1*Δ mutation, suppressed the cold-sensitive growth defect in the *drs2*Δ mutant (Figure 2B). Rcy1p, an F-box protein, binds to the C-terminal cytoplasmic region of Drs2p, and regulates the early endosome-to-TGN retrieval pathway (Furuta *et al.* 2007; Hanamatsu *et al.* 2014). The *cfs1*Δ and *kes1*Δ mutations also suppressed the cold-sensitive growth defect in the *rcy1*Δ mutant (Figure 2B). These results indicate that the *cfs1*Δ and *kes1*Δ mutations suppress the defects in Drs2p-Rcy1p complex-mediated functions.

### The cfs1Δ mutation suppresses defects of growth and membrane trafficking in all of the phospholipid flippase mutants

**Figure 5:**
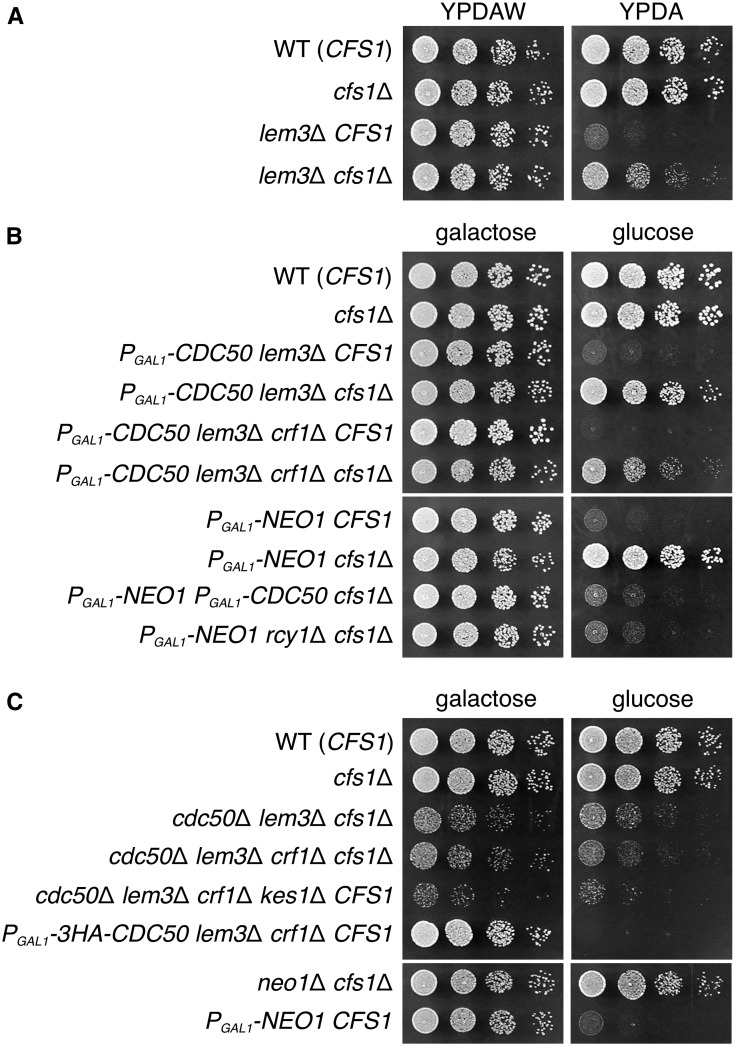
The *cfs1*Δ mutation suppresses the growth defects of all five phospholipid flippase mutants. (A) The *cfs1*Δ mutation suppresses the tryptophan requirement for growth in the *lem3*Δ mutant. Fivefold serial dilutions of exponentially growing cultures were spotted onto YPDAW and YPDA plates, followed by incubation at 30° for 1.5 d. The strains used, all of which are in the *trp1*Δ background, were WT (KKT473), *cfs1*Δ (KKT475), *lem3*Δ (KKT476), and *lem3*Δ *cfs1*Δ (KKT477). (B) The *cfs1*Δ mutation suppresses the growth defect of the Cdc50p-depleted *lem3*Δ *crf1*Δ and Neo1p-depleted cells. Cell spotting was performed on YPGA (galactose) and YPDA (glucose) plates as in (A), and plates were incubated at 30° for 2 d (galactose) or 1.5 d (glucose). The strains used were WT (YKT1066), *cfs1*Δ (YKT2070), *P_GAL1_-3HA-CDC50 lem3*Δ (YKT1890), *P_GAL1_-3HA-CDC50 lem3*Δ *cfs1*Δ (YKT2045), *P_GAL1_-3HA-CDC50 lem3*Δ *crf1*Δ (YKT1120), *P_GAL1_-3HA-CDC50 lem3*Δ *crf1*Δ *cfs1*Δ (YKT2046), *P_GAL1_-NEO1* (YKT2018), *P_GAL1_-NEO1 cfs1*Δ (YKT2085), *P_GAL1_-NEO1 P_GAL1_-3HA-CDC50 cfs1*Δ (YKT2086), and *P_GAL1_-NEO1 rcy1*Δ *cfs1*Δ (YKT2087). (C) The *cfs1*Δ mutation suppresses lethality caused by disruption of *CDC50*, *LEM3*, and *CRF1*, or *NEO1*. The clones containing the indicated disrupted allele were isolated by tetrad dissection of heterozygous diploids, and their cell growth was examined as in (A). Incubation on the YPGA (galactose) and YPDA (glucose) plates was performed at 30° for 2 or 1 d, respectively. The strains used were WT (YKT1066), *cfs1*Δ (YKT2037), *cdc50*Δ *lem3*Δ *cfs1*Δ (YKT2049), *cdc50*Δ *lem3*Δ *crf1*Δ *cfs1*Δ (YKT2050), *cdc50*Δ *lem3*Δ *crf1*Δ *kes1*Δ (YKT2088), *P_GAL1_-3HA-CDC50 lem3*Δ *crf1*Δ (YKT1120), *neo1*Δ *cfs1*Δ (YKT2051), and *P_GAL1_-NEO1* (YKT2018). WT, wild-type; YPDA, yeast extract peptone glucose adenine medium; YPDAW, YPDA supplemented with tryptophan; YPGA, yeast extract peptone galactose adenine medium.

We previously suggested that Lem3p-Dnf1p/Dnf2p are involved in the sorting of high affinity tryptophan permease Tat2p at the TGN; in the *lem3*Δ mutant, Tat2p was not properly transported to the plasma membrane and missorted to the vacuole (Hachiro *et al.* 2013). We examined the effect of the *cfs1*Δ mutation on the requirement of tryptophan for growth in the *lem3*Δ mutant. The *lem3*Δ *trp1*Δ mutant shows a severe growth defect in YPDA medium containing standard concentration of tryptophan (∼100 μg/ml), and requires high concentration of tryptophan (∼300 μg/ml) for growth (Hachiro *et al.* 2013) (Figure 5A). The *cfs1*Δ mutation partially suppressed the growth defect of the *lem3*Δ *trp1*Δ mutant in YPDA, suggesting that the *cfs1*Δ mutation suppresses Tat2p missorting caused by dysfunction of Lem3p-Dnf1p/Dnf2p at the TGN.

We examined whether the *cfs1*Δ mutation can suppress lethality by loss of all Cdc50p family members. Here, we used strains with their chromosomal *CDC50* under the control of the glucose-repressible *GAL1* promoter (referred to as “Cdc50p-depleted”). The *cfs1*Δ mutation suppressed lethality of the Cdc50p-depleted *lem3*Δ *crf1*Δ mutant as well as the Cdc50p-depleted *lem3*Δ mutant (Figure 5B). To confirm that the suppression was not due to the incomplete repression of the *GAL1* promoter, we tried to construct the *cdc50*Δ *lem3*Δ *crf1*Δ *cfs1*Δ mutant by tetrad dissection. We successfully isolated it, although it grew more slowly than the wild type (Figure 5C).

We examined the effect of the *cfs1*Δ mutation on lethality caused by mutations of the other essential phospholipid flippase *NEO1* gene. The *cfs1*Δ mutation suppressed lethality caused by Neo1p-depletion; furthermore, the *neo1*Δ *cfs1*Δ double mutant clone could be isolated by tetrad dissection (Figure 5, B and C). Surprisingly, in contrast with *cdc50*Δ *lem3*Δ *cfs1*Δ and *cdc50*Δ *lem3*Δ *crf1*Δ *cfs1*Δ mutants, the *neo1*Δ *cfs1*Δ mutant exhibited a growth rate similar to that of the wild type, indicating that the *cfs1*Δ mutation is a much more effective suppressor of the *neo1* mutations. However, additional depletion of Cdc50p or the *rcy1*Δ mutation caused severe growth defects in the Neo1p-depleted *cfs1*Δ mutant (Figure 5B), suggesting that the *cfs1*Δ mutation cannot bypass simultaneous loss of all essential phospholipid flippases. We concluded that *cfs1*Δ suppresses growth defects in all five phospholipid flippase mutants.

**Figure 6:**
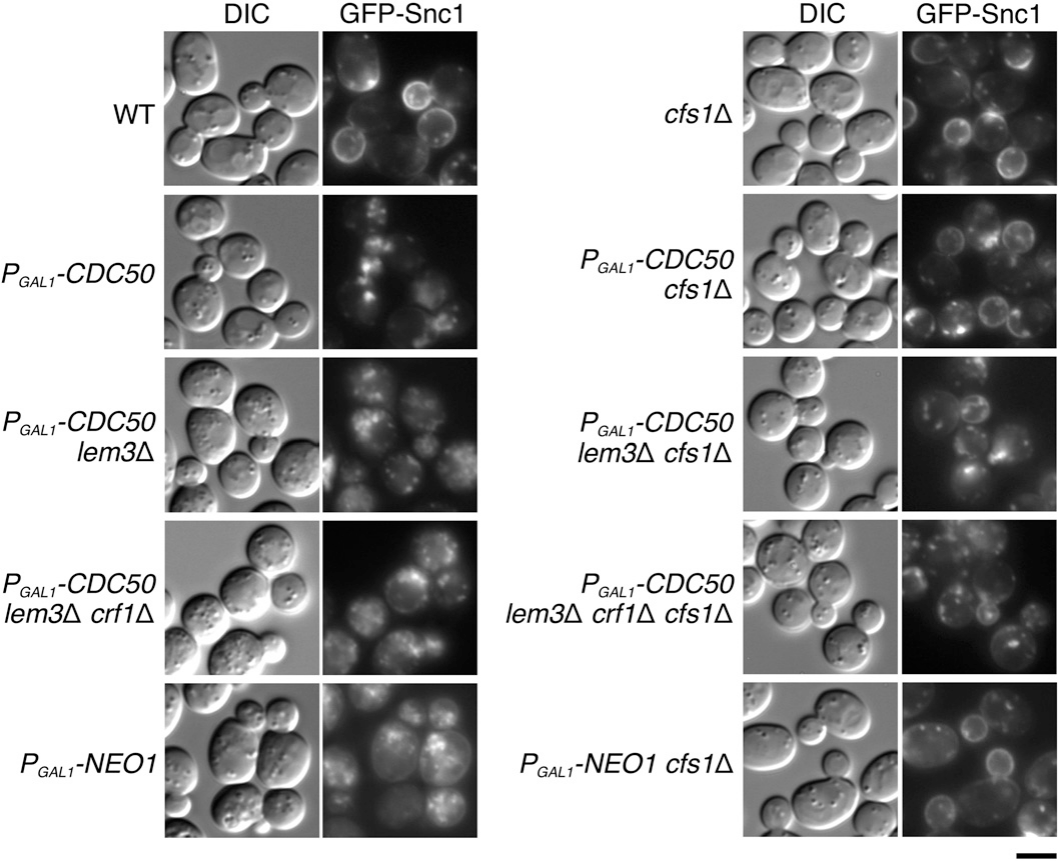
The *cfs1*Δ mutation suppresses the membrane trafficking defect in flippase-deficient mutant cells. Strains expressing *GFP-SNC1* were grown to exponential phase in YPGA medium, washed with YPDA medium, and cultured in YPDA medium at 30° for 12 hr, followed by observation using a fluorescent microscope. The strains used were WT (YKT1523), *cfs1*Δ (YKT2055), *P_GAL1_-3HA-CDC50* (YKT2056), *P_GAL1_-3HA-CDC50 cfs1*Δ (YKT2057), *P_GAL1_-3HA-CDC50 lem3*Δ (YKT2058), *P_GAL1_-3HA-CDC50 lem3*Δ *cfs1*Δ (YKT2059), *P_GAL1_-3HA-CDC50 lem3*Δ *crf1*Δ (YKT2060), *P_GAL1_-3HA-CDC50 lem3*Δ *crf1*Δ *cfs1*Δ (YKT2061), *P_GAL1_-3HA-NEO1* (YKT2062), and *P_GAL1_-3HA-NEO1 cfs1*Δ (YKT2063). All of them carry *P_TPI1_-GFP-SNC1* integrated at the *URA3* locus. Bar, 5 μm. DIC, differential interference contrast; WT, wild-type; GFP, green fluorescent protein; YPDA, yeast extract peptone glucose adenine medium; YPGA, yeast extract peptone galactose adenine medium.

We examined whether the *cfs1*Δ mutation suppressed the defect of membrane trafficking in flippase mutants. Snc1p is a v-SNARE that is transported from the plasma membrane through the early endosome to the TGN and back to the plasma membrane (Lewis *et al.* 2000). We observed its GFP-fused protein to monitor the recycling pathway. In wild-type cells, GFP-Snc1p is mainly localized to polarized sites where exocytosis is actively occurring. Since dysfunction of the Cdc50p family causes the defect in the retrieval pathway from the early endosome to the TGN, GFP-Snc1p displays intracellular accumulation (Saito *et al.* 2004; Furuta *et al.* 2007) (Figure 6). The *cfs1*Δ single mutation did not affect localization of GFP-Snc1p (Figure 6). The *cfs1*Δ mutation suppressed intracellular accumulation of GFP-Snc1p in the Cdc50p-depleted cells; GFP-Snc1p was clearly localized to the polarized plasma membrane sites of the small- or middle-budded cells (∼99% of cells, *n* = 200 cells) (Figure 6). The *cfs1*Δ mutation also partially restored its polarized localization in the Cdc50p-depleted *lem3*Δ and Cdc50p-depleted *lem3*Δ *crf1*Δ mutant cells, both of which exhibited more severe GFP-Snc1p accumulation compared to that of Cdc50p-depleted cells; GFP-Snc1p was slightly localized to the polarized plasma membrane sites of the middle-budded cells (∼90% of cells, *n* = 200 cells), but intracellular accumulation of GFP-Snc1p remained in many cells (∼40%, *n* = 200 cells) (Figure 6). The *neo1* mutations cause defects in membrane trafficking within and from the endosomal/Golgi system (Hua and Graham 2003; Wicky *et al.* 2004). GFP-Snc1p accumulated intracellularly in the Neo1p-depleted cells, whereas it displayed almost normal localization in the Neo1p-depleted *cfs1*Δ cells (100%, *n* = 200 cells) (Figure 6) and even in the *neo1*Δ *cfs1*Δ mutant cells (100%, *n* = 200 cells) (Figure S1), consistent with complete suppression of growth defects. These results suggest that the *cfs1*Δ mutation suppresses membrane trafficking defects in phospholipid flippase mutants.

### Cfs1p functions in the trafficking pathway from endosomes to the TGN

**Figure 7:**
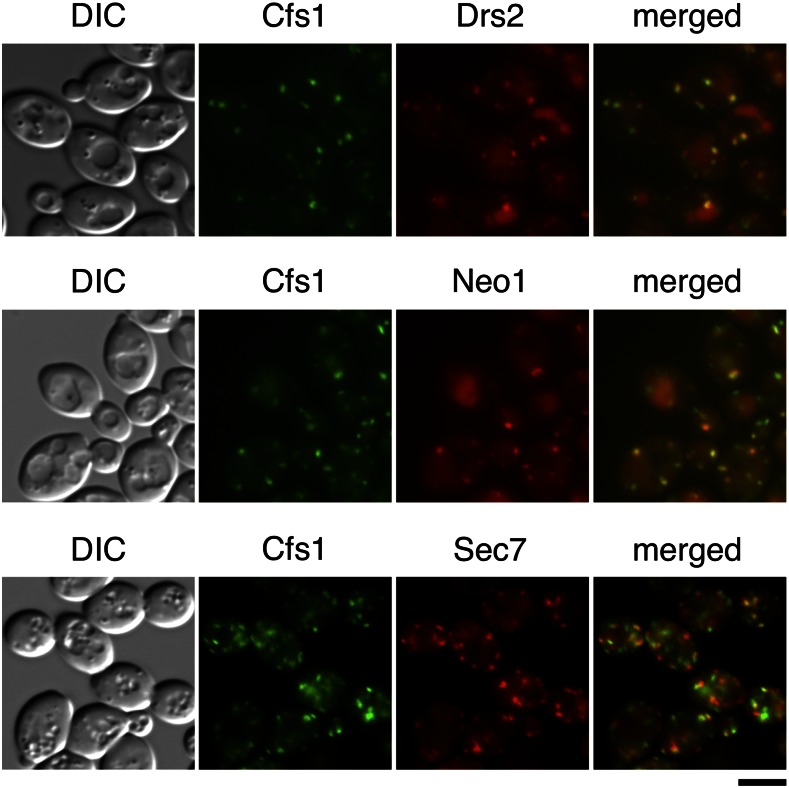
Cfs1p is partially colocalized with Drs2p, Neo1p, and Sec7p. Strains expressing *CFS1-EGFP* and *DRS2-mRFP1*, *NEO1-mRFP1*, or *SEC7-mRFP1* were grown to exponential phase in YPDA medium at 30°, followed by fluorescent microscopic observation after fixation with 0.2% (Drs2p-mRFP1 and Neo1p-mRFP1) or 2% (Sec7p-mRFP1) of formaldehyde. Obtained images were merged to compare the two signal patterns. Homologous diploid strains were used for better visualization. The strains used were *CFS1-EGFP DRS2-mRFP1* (YKT2093), *CFS1-EGFP NEO1-mRFP1* (YKT2094), and *CFS1-EGFP SEC7-mRFP1* (YKT2111). Bar, 5 μm. DIC, differential interference contrast; EGFP, enhanced green fluorescent protein; mRFP1, monomeric red fluorescent protein 1; YPDA, yeast extract peptone glucose adenine medium.

Cellular localization of Cfs1p was examined by its C-terminal EGFP fusion. Cfs1p-EGFP was functional, because it did not suppress the cold-sensitive growth defect in the *cdc50*Δ mutant (Figure S2). Cfs1p-EGFP displayed intracellular punctate structures (Figure 7). Its localization was compared with that of C-terminally mRFP1-fused Drs2p and Neo1p, both of which are localized to the TGN and endosomal structures (Hua *et al.* 2002; Hua and Graham 2003; Saito *et al.* 2004; Wicky *et al.* 2004). Cfs1p-EGFP was partially colocalized with Drs2p-mRFP1 and Neo1p-mRFP1 (Figure 7); 34 and 61% of Cfs1p-EGFP-positive structures were colocalized with Drs2p-mRFP1-positive (*n* = 229) and Neo1p-mRFP1-positive structures (*n* = 236), respectively, whereas 63% of Drs2p-mRFP1-positive and 64% of Neo1p-mRFP1-positive structures were colocalized with Cfs1p-EGFP-positive structures (*n* = 225 and 223, respectively). We also compared localization of Cfs1p-EGFP with that of C-terminally mRFP1-fused Sec7p, a TGN marker (Franzusoff *et al.* 1991). Cfs1p-EGFP was partially colocalized with Sec7p-mRFP1 (Figure 7); 29% of Cfs1p-positive structures were colocalized with Sec7p-mRFP1-positive structures (*n* = 214), whereas 45% of Sec7p-mRFP1-positive structures were colocalized with Cfs1p-EGFP-positive structures (*n* = 268). These results suggest that Cfs1p functions at the TGN and/or endosomes like flippases. We previously showed that Cdc50p-Drs2p was confined to the plasma membrane upon blockade of endocytosis, indicating that Cdc50p-Drs2p cycles between the exocytic and endocytic pathways (Saito *et al.* 2004). A disruption mutation of *VRP1*, the WASP-interacting protein involved in endocytosis (Munn *et al.* 1995), did not affect localization of Cfs1p-EGFP (Figure S3), suggesting that this protein remains at TGN/endosomal membranes.

**Figure 8:**
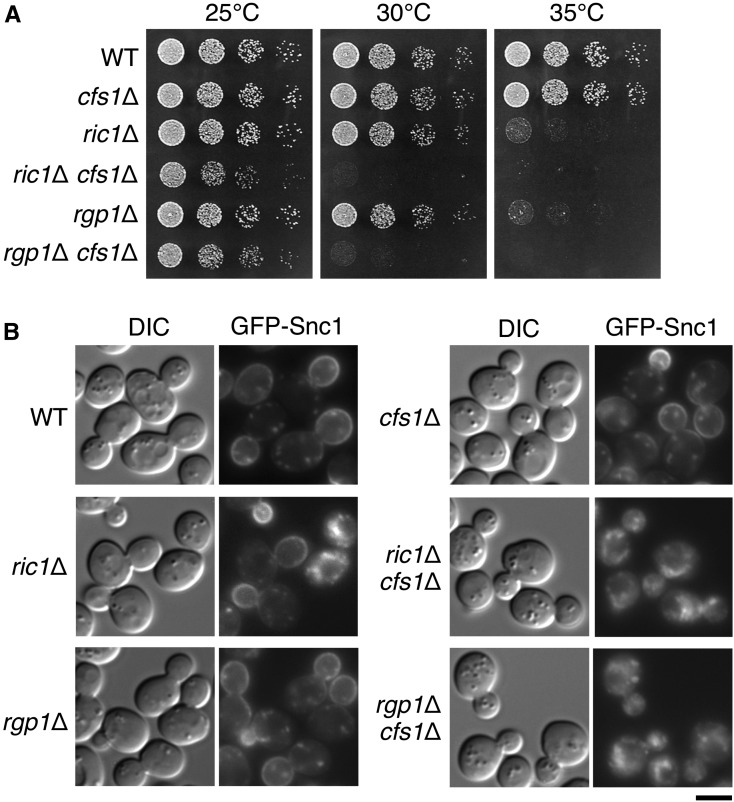
Synthetic defects in cell growth and Snc1p v-SNARE trafficking between the *cfs1*Δ mutation and disruption mutations of the Ric1p/Rgp1p GEF complex for Ypt6p rab GTPase. (A) The *cfs1*Δ mutation exacerbates temperature-sensitive growth of *ric1*Δ and *rgp1*Δ mutants. Fivefold serial dilutions of exponentially growing cultures were spotted onto YPDA plates, followed by incubation at 25° for 2 d, or at 30 or 35° for 1.5 d. The strains used were WT (YKT1066), *cfs1*Δ (YKT2037), *ric1*Δ (YKT2020), *ric1*Δ *cfs1*Δ (YKT2033), *rgp1*Δ (YKT2022), and *rgp1*Δ *cfs1*Δ (YKT2034). (B) The *cfs1*Δ mutation exhibits synthetic defects in GFP-Snc1p transport with the *ric1*Δ and *rgp1*Δ mutations. Strains expressing *GFP-SNC1* were grown to exponential phase in YPDA medium at 25°, transferred to 30°, and cultured for 4 hr, followed by observation using a fluorescent microscope. The strains used were WT (YKT1523), *cfs1*Δ (YKT2055), *ric1*Δ (YKT2095), *ric1*Δ *cfs1*Δ (YKT2096), *rgp1*Δ (YKT2097), and *rgp1*Δ *cfs1*Δ (YKT2098). All of them carry *P_TPI1_-GFP-SNC1* integrated at the *URA3* locus. Bar, 5 μm. DIC, differential interference contrast; GEF guanine nucleotide exchange factor; GFP, green fluorescent protein; GTPase, guanosine triphosphatase; v-SNARE, vesicle soluble NSF attachment protein receptor; WT, wild-type; YPDA, yeast extract peptone glucose adenine medium.

Previously, synthetic genetic array (SGA) analyses showed that the *ymr010w*Δ mutation causes synthetic growth defects with the *ypt6*Δ and *ric1*Δ mutations (Tong *et al.* 2004). Ypt6p, the yeast counterpart of mammalian Rab6 GTPase, functions in endosome-to-TGN and intra-Golgi retrograde transport (Luo and Gallwitz 2003), together with its guanine nucleotide exchange factor (GEF), the Ric1p/Rgp1p complex (Siniossoglou *et al.* 2000). We manually confirmed the synthetic genetic interaction of *cfs1*Δ with *ric1*Δ or *rgp1*Δ. The *ric1*Δ and *rgp1*Δ mutants show temperature-sensitive growth (Mizuta *et al.* 1997; Siniossoglou *et al.* 2000) (Figure 8A). They grew as well as the wild-type strain at 30°, whereas the *cfs1*Δ mutation caused severe growth defects in these mutants at this temperature (Figure 8A). Rgp1p is required for recycling of Snc1p at 35° (Panek *et al.* 2000) (data not shown). However, at 30°, most cells of *ric1*Δ and *rgp1*Δ mutants (80–90%, *n* = 200 cells) showed normal polarized localization of GFP-Snc1p (Figure 8B). The *cfs1*Δ mutation led to intracellular accumulation of GFP-Snc1p in these mutants at this temperature (∼90%, *n* = 200 cells) (Figure 8B). These results suggest that Cfs1p is involved in endosome-to-TGN transport in a manner redundant with Ric1p/Rgp1p and Ypt6p. Considering that the *cfs1*Δ mutation suppresses flippase mutations, these results suggest that Cfs1p functions antagonistically to phospholipid flippases in the endocytic recycling pathway.

### Cfs1p may regulate asymmetrical distribution of phospholipids

**Figure 9:**
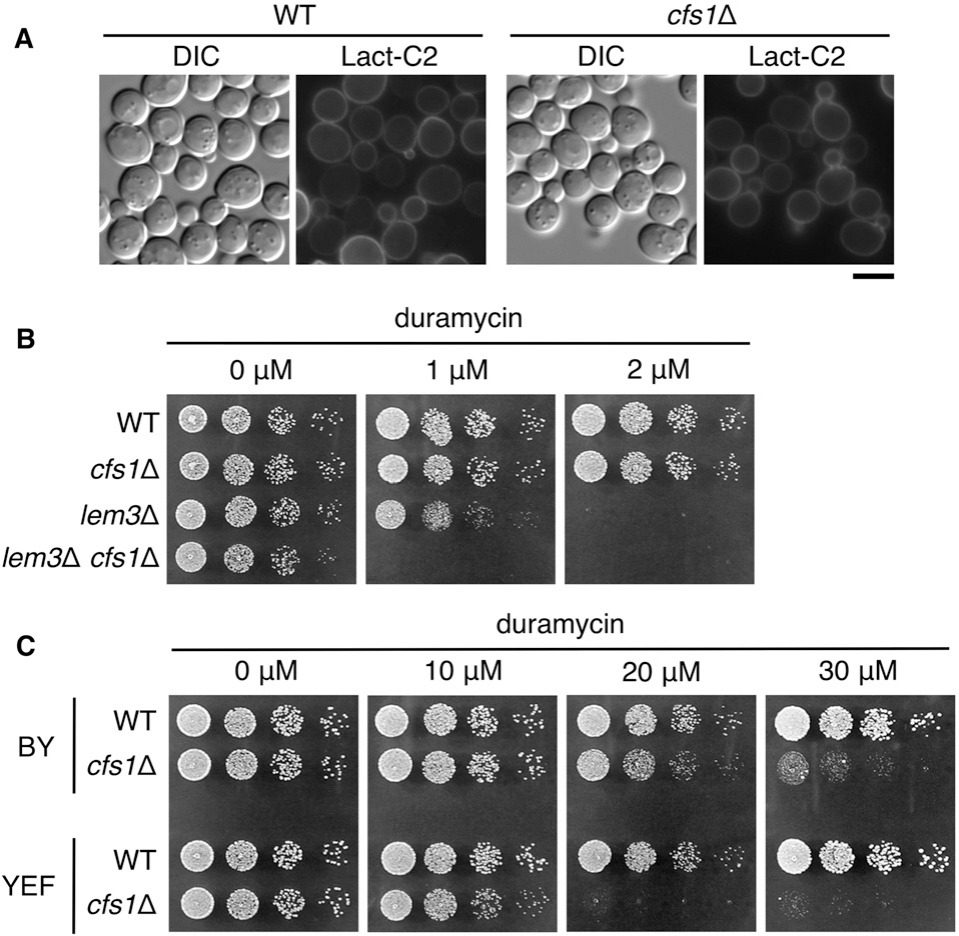
Cfs1p may be involved in asymmetric distribution of PE. (A) The *cfs1*Δ mutation does not affect localization of GFP-Lact-C2. Strains harboring pRS416-P_GPD_-GFP-Lact-C2 were grown to exponential phase in SD-Ura medium at 30°, followed by observation using a fluorescent microscope. The strains used were WT (YKT1066) and *cfs1*Δ (YKT2037). Bar, 5 μm. (B) The *cfs1*Δ mutation enhances duramycin sensitivity of the *lem3*Δ mutant. Fivefold serial dilutions of exponentially growing cultures were spotted onto YPDA plates containing duramycin at the indicated concentration, followed by incubation at 30° for 1 d. The strains used were WT (YKT1066), *cfs1*Δ (YKT2070), *lem3*Δ (YKT715), and *lem3*Δ *cfs1*Δ (YKT2099). (C) The *cfs1*Δ mutant is sensitive for growth to duramycin at a high concentration. Cell spotting was performed as in (B), and plates were incubated at 30° for 1 d (0, 10, and 20 μM) or 2 d (30 μM). The strains used were WT (KKT61) and *cfs1*Δ (KKT478) that were derived from BY4743 (BY), and WT (YKT1066) and *cfs1*Δ (YKT2070) that were derived from YEF473 (YEF). DIC, differential interference contrast; GFP, green fluorescent protein; PE, phosphatidylethanolamine; SD, synthetic glucose; WT, wild-type; YPDA, yeast extract peptone glucose adenine medium.

We next wanted to examine whether the *cfs1*Δ mutation affected phospholipid asymmetry. The GFP-tagged C2 domain of lactadherin (GFP-Lact-C2) specifically binds to PS, and then enables visualization of endogenous PS in the cytoplasmic leaflet of membranes (Yeung *et al.* 2008). In wild-type cells, GFP-Lact-C2 was localized to the plasma membrane, but not to any other organelle including the TGN and endosomes (Yeung *et al.* 2008) (Figure 9A). Considering that Cfs1p may antagonize the flippase function, the *cfs1*Δ mutation may cause PS exposure to the cytoplasmic leaflet of endosomal/TGN membranes. However, the *cfs1*Δ mutant did not display GFP-Lact-C2 localization to intracellular organelles (Figure 9A). Because it is possible that the effect of the *cfs1*Δ mutation is undetectable by this probe, we next attempted a more sensitive test using duramycin, a tetracyclic peptide that binds to PE (Navarro *et al.* 1985). The *lem3*Δ mutant cells expose PE in the outer leaflet of the plasma membrane and then exhibit sensitivity for growth to duramycin. The *cfs1*Δ mutation exacerbated duramycin-sensitive growth in the *lem3*Δ mutant (Figure 9B). Furthermore, the *cfs1*Δ single mutant exhibited sensitivity to a high concentration of duramycin (Figure 9C) in two different strain backgrounds, BY4741 (Brachmann *et al.* 1998) and YEF473 (Bi and Pringle 1996). We confirmed that these duramycin sensitivities were complemented by *CFS1* expression from a centromeric plasmid (Figure S4). As described above, Cfs1p was localized to endosomal/Golgi membranes and was not transported to the plasma membrane. These results suggest that the *cfs1*Δ mutation indirectly affects phospholipid asymmetry of the plasma membrane, probably through membrane transport between endosomal/Golgi membranes and the plasma membrane. Cfs1p may be involved in regulating the asymmetric distribution of phospholipids in endosomal/Golgi membranes.

### The neo1Δ cfs1Δ mutant displays a growth defect to high sodium salt

**Figure 10:**
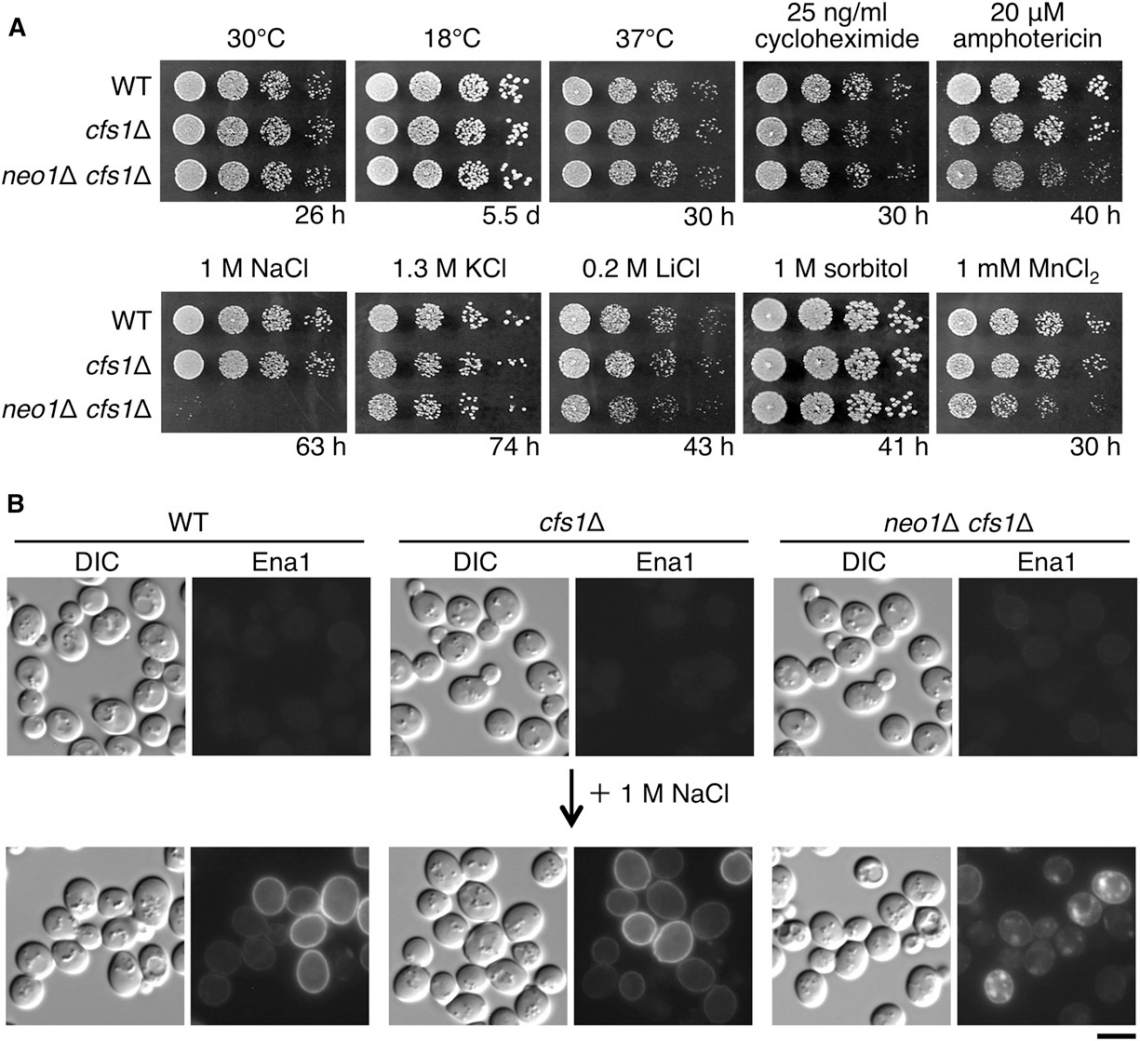
The *neo1*Δ *cfs1*Δ mutant exhibits a growth defect to high sodium salt. (A) The *neo1*Δ *cfs1*Δ mutant shows NaCl-sensitive growth. Fivefold serial dilutions of exponentially growing cultures were spotted onto YPDA plates supplemented with indicated chemicals or drugs, followed by incubation at 30° for the indicated time. Cell growth was also examined at 18 or 37°. The strains used were WT (YKT1066), *cfs1*Δ (YKT2037), and *neo1*Δ *cfs1*Δ (YKT2051). (B) The *neo1*Δ *cfs1*Δ mutant is defective in localization of the Ena1p sodium export pump to the plasma membrane. Strains harboring the *ENA1-GFP* allele were grown to exponential phase in YPDA medium (upper panels), washed with YPDA medium containing 1 M NaCl, and cultured in the same medium at 30° for 3 hr (lower panels). Cells were harvested and suspended in SD medium containing 1 M NaCl, followed by observation using a fluorescent microscope. The strains used were WT (YKT2100), *cfs1*Δ (YKT2101), and *neo1*Δ *cfs1*Δ (YKT2102). The GFP gene was fused to the C-terminus of the chromosomal *ENA1* gene in these strains. Bar, 5 μm. DIC, differential interference contrast; GFP, green fluorescent protein; PE, phosphatidylethanolamine; SD, synthetic glucose; WT, wild-type; YPDA, yeast extract peptone glucose adenine medium.

Suppression of the lethality of the *neo1*Δ mutant by the *cfs1*Δ mutation was so complete that the *neo1*Δ *cfs1*Δ mutant grew like wild-type cells at 30, 18, and 37° (Figure 10A). Finding a condition that renders the *neo1*Δ *cfs1*Δ mutant defective for growth may give us a clue why these two genes evolved. We tested growth of the *neo1*Δ *cfs1*Δ mutant in various stress conditions. The acidic condition (pH 3.0) inhibited growth only slightly, but the alkaline condition (pH 8.0) did not (Figure S5). We also tested some compounds including cycloheximide, amphotericin B (an ergosterol-binding polyene antibiotic), and MnCl_2_, but again cell growth was not affected (Figure 10A). When supplemented with a high concentration of salt, we found that 1 M NaCl strongly inhibited growth, but 0.2 M LiCl only slightly inhibited growth, and 1.3 M KCl did not affect growth (Figure 10A), indicating that this mutant exhibits sensitivity specific to a high concentration of sodium cations. This sensitivity was not caused by hyperosmotic stress, because supplementation with 1 M sorbitol did not affect growth of the *neo1*Δ *cfs1*Δ mutant (Figure 10A). Ena P-type ATPases function for efflux of sodium cations at the plasma membrane (Ariño *et al.* 2010). We examined whether the sodium sensitivity of the *neo1*Δ *cfs1*Δ mutant was due to a defect in production or localization of the Ena1 sodium export protein by observation of chromosomally GFP-tagged Ena1p. In cells cultured in standard rich medium, the signal of Ena1p-GFP was hardly detectable (Figure 10B, upper). When supplemented with 1 M NaCl for 3 hr, Ena1p-GFP displayed exclusive localization to the plasma membrane in wild-type and *cfs1*Δ cells. In contrast, *neo1*Δ *cfs1*Δ cells showed intracellular accumulation of Ena1p (∼80%, *n* = 200 cells) in addition to localization at the plasma membrane (Figure 10B, lower), suggesting that some population of Ena1p was mistargeted in this mutant. These results suggest that the Neo1p/Cfs1p system is involved in the transport of Ena proteins in sodium stress conditions.

### Cfs1p and Kes1p play distinct roles in flippase-mediated functions

**Figure 11:**
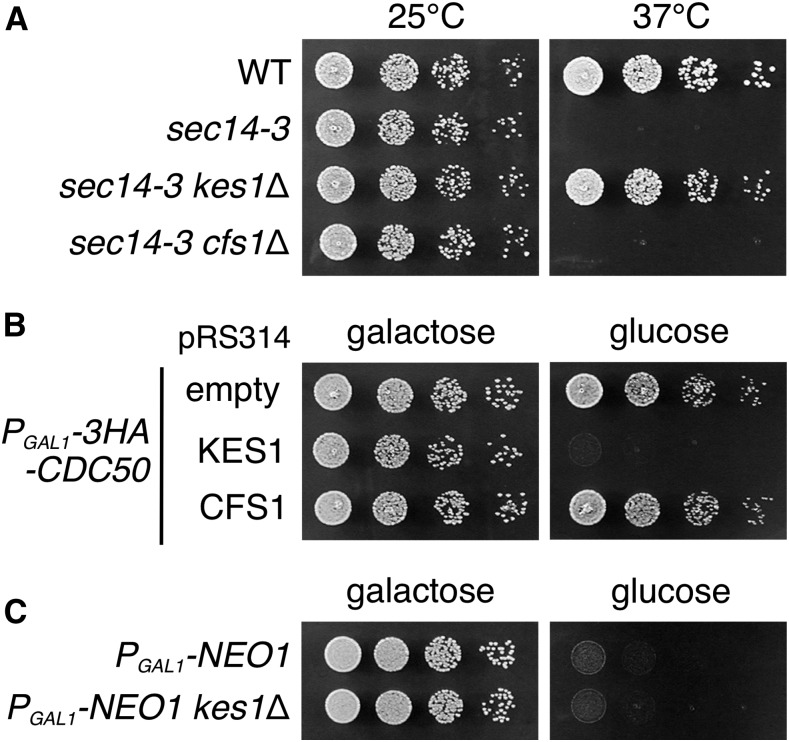
*CFS1* and *KES1* exhibit distinct genetic interactions. (A) The *cfs1*Δ mutation cannot suppress temperature-sensitive growth of the *sec14-3* mutant. Fivefold serial dilutions of exponentially growing cultures were spotted onto YPDA plates, followed by incubation at 25 and 37° for 2 d. The strains used were WT (YKT1066), *sec14-3* (YKT2074), *sec14-3 kes1*Δ (YKT2075), and *sec14-3 cfs1*Δ (YKT2076). (B) An additional dose of *KES1*, but not of *CFS1*, inhibits growth of Cdc50-depleted cells. Cell spotting was performed on SGA-Trp (galactose) and SDA-Trp (glucose) plates as in (A), and plates were incubated at 30° for 2 d. The strain used was *P_GAL1_-3HA-CDC50* (YKT1638), which contains pRS314 plasmid harboring the indicated gene. (C) The *kes1*Δ mutation cannot suppress lethality of Neo1p-depleted cells. Cell spotting was performed on YPGA (galactose) and YPDA (glucose) plates as in (A), and plates were incubated at 30° for 2 d (galactose) or 1.5 d (glucose). The strains used were *P_GAL1_-NEO1* (YKT2018) and *P_GAL1_-NEO1 kes1*Δ (YKT2069). YPDA, yeast extract peptone glucose adenine medium; YPGA, yeast extract peptone galactose adenine medium; SGA, synthetic galactose casamino acids medium; SDA, synthetic glucose casamino acids medium; WT, wild-type.

In our screen, the *kes1* mutation was also identified as a strong suppressor for the *cdc50*Δ mutant. Kes1p, also known as Osh4p, is a member of the oxysterol-binding protein (OSBP) homolog (Osh) family (Jiang *et al.* 1994; Beh *et al.* 2001). To examine whether Cfs1p and Kes1p have similar functions, we compared genetic interactions that *CFS1* and *KES1* exhibit. Loss of Kes1p has been shown to suppress defects in cell growth, phosphatidylinositol (PI) levels, and exocytosis in the mutant of the PI/PC transfer protein Sec14p (Fang *et al.* 1996; Li *et al.* 2002). In contrast to the *kes1*Δ mutation, the *cfs1*Δ mutation did not suppress temperature-sensitive growth of the *sec14-3* mutant (Figure 11A). Overexpression of *KES1* was shown to decrease the level of PI-4-phosphate [PI(4)P] (LeBlanc and McMaster 2010). As shown in Figure 11B, additional dosage of *KES1* on a single-copy plasmid inhibited growth of Cdc50p-depleted cells, consistent with the requirement of PI(4)P for Drs2p activity (Natarajan *et al.* 2009) and a negative role of Kes1p for Drs2p flippase activity (Muthusamy *et al.* 2009). In contrast, additional expression of *CFS1* from a single-copy plasmid (Figure 11B) or even a multi-copy plasmid (Figure S6) did not affect growth of Cdc50p-depleted cells. We next showed that, in contrast to the *cfs1*Δ mutation (Figure 5B), the *kes1*Δ mutation did not suppress lethality of Neo1p-depleted cells (Figure 11C). These results suggest that Cfs1p is involved in flippase-mediated functions in a manner different from that of Kes1p.

## Discussion

### Isolation of suppressor mutations of the cdc50Δ mutation

In this study, we performed transposon-insertion mutagenesis to find mutations that suppress the cold-sensitive growth defect in the *cdc50*Δ mutant, and isolated several genes in addition to the previously identified *kes1* mutation (Muthusamy *et al.* 2009). *FUN26* and *PLB3* were identified as weak suppressors. *FUN26* encodes a vacuolar membrane-localized transporter for nucleoside and nucleobase (Vickers *et al.* 2000) or nicotinamide (Lu and Lin 2011). Interestingly, its deletion was identified in a screen for mutants that overproduce and excrete inositol (Opi) into the growth medium in the absence of inositol and choline (Opi^−^ phenotype) (Hancock *et al.* 2006). Opi1p, which was identified in the original study of this screen (Greenberg *et al.* 1982), is a repressor of the phospholipid biosynthesis genes. The Opi^−^ phenotype of the *fun26* mutant was suppressed by the addition of choline into the medium, as were mutants of *CHO2* and *OPI3* encoding enzymes that catalyze PC biosynthesis, suggesting that Fun26p is involved in this pathway. Fun26p might be involved in the phospholipid flippase functions through regulation of PC biosynthesis.

Plb3p is a phospholipase B working in the periplasmic space, and hydrolyzes PS and PI. In addition, it was shown to exhibit transacylase activity *in vitro*, catalyzing the synthesis of PI from two molecules of lyso-PI (Merkel *et al.* 1999). The *plb3* mutation may suppress defects in phospholipid flippase mutants by indirectly changing phospholipid composition or the distribution of intracellular membranes.

### Cfs1p is involved in membrane trafficking at endosomal/TGN membranes

Previous SGA analysis revealed a synthetic growth defect of *cfs1*Δ and the *pik1-101* allele (Demmel *et al.* 2008). Pik1p, a PI 4-kinase at the TGN, is involved in various membrane trafficking pathways including TGN-to-plasma membrane, TGN-to-vacuole, and transport between the TGN and the early endosome (Strahl and Thorner 2007). Cfs1p was partially colocalized with Drs2p and Neo1p to endosomal/TGN membranes (Figure 7). Consistent with the functions of Drs2p and Neo1p in the endocytic recycling pathway (Furuta *et al.* 2007; Takeda *et al.* 2014), *cfs1*Δ exhibited synthetic defects in growth and Snc1p transport with *ric1*Δ and *rgp1*Δ mutations (Figure 8). Mammalian RAG1AP1 (SWEET1) regulates the trafficking of the TRPV2 ion channel to the plasma membrane via physical interaction (Stokes *et al.* 2005). The involvement of the PQ-loop family in membrane trafficking by functioning as cargo receptors is an interesting model based on the similarity of predicted structures between PQ-loop proteins and the KDEL receptor (Saudek 2012). On the other hand, here we reveal a novel function of Cfs1p, which seems to have an antagonistic function against phospholipid flippases.

### Is Cfs1p a regulator of phospholipid asymmetry?

Cfs1p belongs to the PQ-loop transporter family, which includes the SWEET sugar transporter and mitochondrial pyruvate carrier (MPC) in addition to lysosomal/vacuolar amino acid and cystine transporters. Ypq1p/Ypq2p/Ypq3p, which are yeast PQ-loop proteins, are indicated to export and import basic amino acids at the vacuole (Jézégou *et al.* 2012; Sekito *et al.* 2014; Manabe *et al.* 2016); furthermore, SWEETs are also indicated to transport sugars bidirectionally (Eom *et al.* 2015), although a precise transport mechanism has not been elucidated. Since these characterized transporters transport amino acids or sugars, Cfs1p may similarly transport some small molecule. We previously showed that inositol depletion from culture medium suppressed defects in both growth and membrane trafficking in flippase mutants (Yamagami *et al.* 2015). Thus, the *cfs1*Δ mutation might suppress flippase mutations by decreasing the cytoplasmic inositol level. Inositol is an essential nutrient for growth in yeast; in the absence of *INO1* responsible for *de novo* inositol biosynthesis, yeast cell growth relies on inositol in culture medium (Henry *et al.* 2012). However, the *cfs1*Δ mutation did not affect cell growth in the *ino1*Δ mutant (data not shown), suggesting that Cfs1p does not play a major role in controlling the cytoplasmic concentration of inositol.

One fascinating possibility is that Cfs1p regulates transbilayer movement of phospholipids. Genetic interactions presented here suggest that Cfs1p antagonizes flippase functions; Cfs1p might regulate floppase or scramblase activity. Since phospholipid flip and flop antagonize each other, these activities should be strictly regulated in a spatiotemporal manner. In the plasma membrane, PS is enriched in the cytoplasmic leaflet, not in the exoplasmic leaflet, and this topology appears to be maintained in endocytic vesicles (Pranke *et al.* 2011; Sun and Drubin 2012). Thus, PS needs to be transported to the luminal leaflet upon fusion with early endosomes, because PS is a favorable substrate of Drs2p flippase for vesicle formation (Baldridge and Graham 2012). Cfs1p is likely a candidate protein or a regulatory protein for the floppase/scramblase activity. In this scenario, PS remains to be exposed in the cytoplasmic leaflet of early endosomes in the *cfs1*Δ mutant. Although we could not detect PS in intracellular membranes in the *cfs1*Δ mutant with GFP-Lact-C2 (Figure 9A), the level of PS exposed on early endosomes may be too low to be detected by GFP-Lact-C2. If PS plays some role in vesicle biogenesis (*e.g.*, recruitment of a clathrin adaptor), the *cfs1*Δ mutation would suppress flippase mutations. However, unregulated transbilayer phospholipid distribution will lead to defective vesicle trafficking. In fact, the *cfs1*Δ mutation and *ric1*Δ/*rgp1*Δ mutations exhibited synthetic defects in Snc1p trafficking (Figure 8B), and the Ena1p sodium efflux pump was not properly transported to the plasma membrane in the *neo1*Δ *cfs1*Δ mutant (Figure 10).

Consistent with our hypothesis described above, the *cfs1*Δ mutation exacerbated duramycin-sensitive growth of the *lem3*Δ mutant cells and by itself caused growth defects at a high concentration of duramycin (Figure 9). Since Cfs1p was localized to endosomal/TGN membranes (Figure 7), the effects on PE asymmetry at the plasma membrane seem to be mediated by vesicular trafficking. Cfs1p may directly regulate phospholipid asymmetry, but it is also possible that Cfs1p regulates the localization or activity of an unknown floppase or scramblase. Phospholipid scramblase activity was unexpectedly detected in a G protein-coupled receptor. Goren *et al.* (2014) demonstrated that, upon reconstitution into vesicles, rhodopsin facilitated rapid scrambling of phospholipid probes in an ATP-independent manner. Similar biochemical experiments are needed to clarify the enzymatic activity of Cfs1p.

## Supplementary Material

Supplemental material is available online at www.g3journal.org/lookup/suppl/doi:10.1534/g3.116.035238/-/DC1.
