# Supplementary material for: Cfs1p, a Novel Membrane Protein in the PQ-Loop Family, Is Involved in Phospholipid Flippase Functions in Yeast
Source: G3 (Bethesda). 2016 Nov 8;7(1):179–92. doi: 10.1534/g3.116.035238 (PMC5217107; doi:10.1534/g3.116.035238)
Supplement: Supplementary file 1 [file 179FigureS1.pdf]

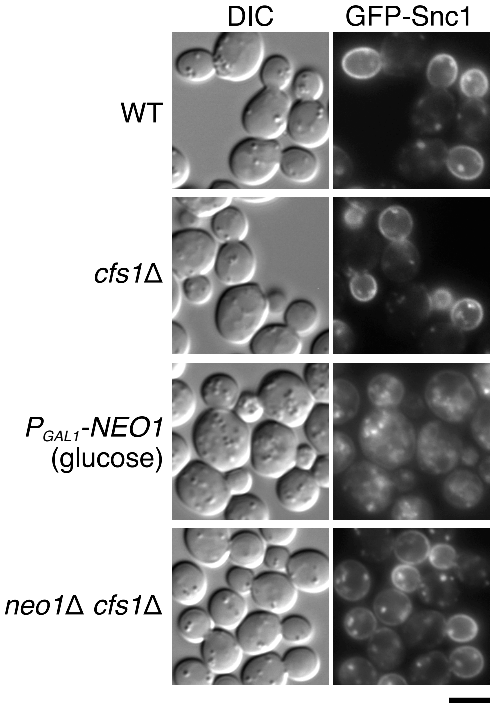

Figure S1 The *neo1*Δ *cfs1*Δ mutant shows almost normal localization of GFP-Snc1p. Strains expressing *GFP-SNC1* were grown to exponential phase in YPDA medium at 30°C, followed by observation using a fluorescent microscope. The P_{GAL1}-*3HA-NEO1* strain was cultured as in Figure 6. The strains used were wild type (YKT1523), *cfs1*Δ (YKT2055), P_{GAL1}-*3HA-NEO1* (YKT2062), and *neo1*Δ *cfs1*Δ (YKT2104). All of them carry P_{TPI1}-*GFP-SNC1* integrated at the *URA3* locus. Bar, 5 μm.
