# Supplementary material for: Cfs1p, a Novel Membrane Protein in the PQ-Loop Family, Is Involved in Phospholipid Flippase Functions in Yeast
Source: G3 (Bethesda). 2016 Nov 8;7(1):179–92. doi: 10.1534/g3.116.035238 (PMC5217107; doi:10.1534/g3.116.035238)
Supplement: Supplementary file 2 [file 179FigureS2.pdf]

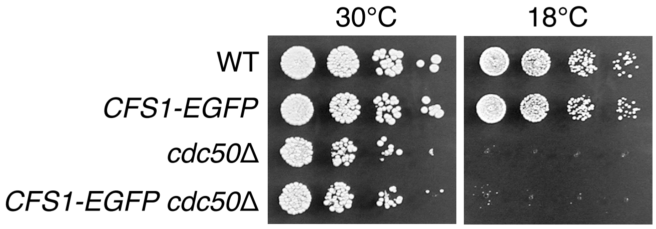

Figure S2 Cfs1p-EGFP is functional. Five-fold serial dilutions of exponentially growing cultures were spotted onto YPDA plates, followed by incubation at 30°C for 3 days or at 18°C for 5 days. *CFS1-EGFP* did not suppress the cold-sensitive growth in the *cdc50*Δ mutant. The strains used were wild type (WT, YKT38), *CFS1-EGFP* (YKT2079), *cdc50*Δ (YKT249), and *CFS1-EGFP cdc50*Δ (YKT2105).
