# Supplementary material for: Cfs1p, a Novel Membrane Protein in the PQ-Loop Family, Is Involved in Phospholipid Flippase Functions in Yeast
Source: G3 (Bethesda). 2016 Nov 8;7(1):179–92. doi: 10.1534/g3.116.035238 (PMC5217107; doi:10.1534/g3.116.035238)
Supplement: Supplementary file 3 [file 179FigureS3.pdf]

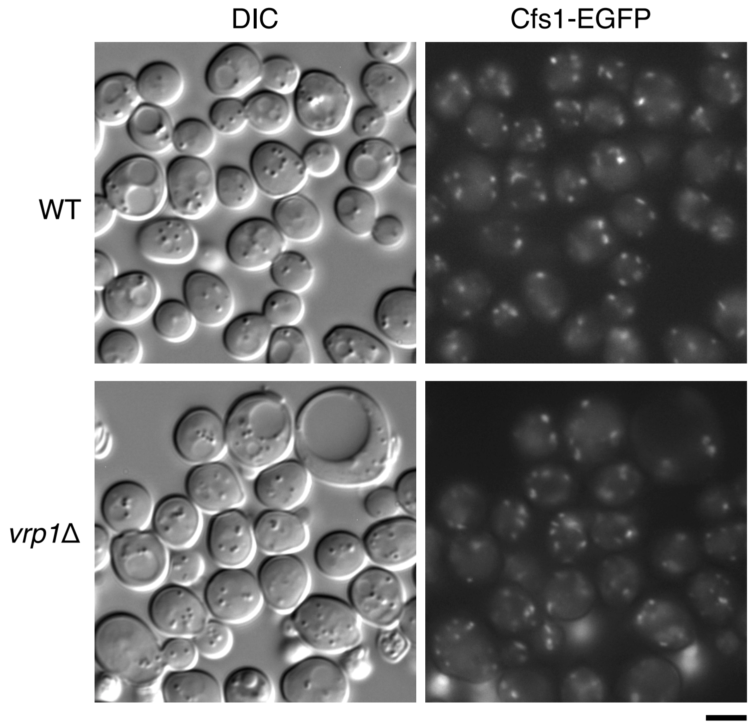

Figure S3 The *vrp1Δ* mutation does not affect localization of Cfs1p-EGFP. Strains expressing *CFS1-EGFP* were grown to exponential phase in YPDA medium at 25°C, followed by observation using a fluorescent microscope. The strains used were *CFS1-EGFP* (WT, YKT2079) and *CFS1-EGFP vrp1Δ* (YKT2106). Bar, 5 μm.
