# Supplementary material for: Cfs1p, a Novel Membrane Protein in the PQ-Loop Family, Is Involved in Phospholipid Flippase Functions in Yeast
Source: G3 (Bethesda). 2016 Nov 8;7(1):179–92. doi: 10.1534/g3.116.035238 (PMC5217107; doi:10.1534/g3.116.035238)
Supplement: Supplementary file 4 [file 179FigureS4.pdf]

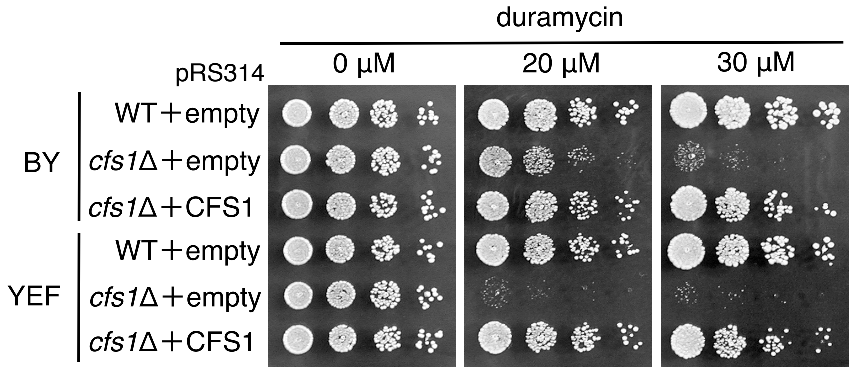

Figure S4 Duramycin-sensitive growth of the *cfs1*Δ mutants was complemented by the centromeric plasmid containing the *CFS1* gene. Five-fold serial dilutions of exponentially growing cells, which harbor pRS314 (empty) or pRS314-CFS1, were spotted onto YPDA plates containing duramycin at the indicated concentration, followed by incubation at 30°C for 1.5 days (0 and 20 μM) or 2 days (30 μM). The strains used were wild type (WT, KKT473) and *cfs1*Δ (KKT475) that were derived from BY4743 (BY), and wild type (WT, YKT38) and *cfs1*Δ (YKT2064) that were derived from YEF473 (YEF).
