# Supplementary material for: Cfs1p, a Novel Membrane Protein in the PQ-Loop Family, Is Involved in Phospholipid Flippase Functions in Yeast
Source: G3 (Bethesda). 2016 Nov 8;7(1):179–92. doi: 10.1534/g3.116.035238 (PMC5217107; doi:10.1534/g3.116.035238)
Supplement: Supplementary file 5 [file 179FigureS5.pdf]

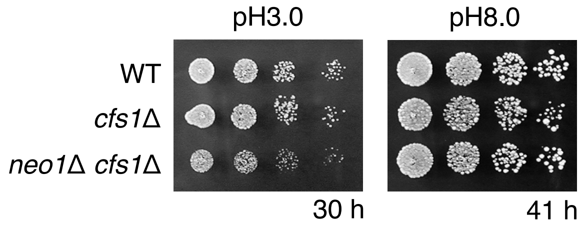

Figure S5 Effect of pH in the medium on the growth rate of the *neo1*Δ *cfs1*Δ mutant. Five-fold serial dilutions of exponentially growing cultures were spotted onto YPDA plates adjusted to indicated pH, followed by incubation at 30°C for the indicated time. The strains used were wild type (WT, YKT1066), *cfs1*Δ (YKT2037), and *neo1*Δ *cfs1*Δ (YKT2051).
