# Supplementary material for: Cfs1p, a Novel Membrane Protein in the PQ-Loop Family, Is Involved in Phospholipid Flippase Functions in Yeast
Source: G3 (Bethesda). 2016 Nov 8;7(1):179–92. doi: 10.1534/g3.116.035238 (PMC5217107; doi:10.1534/g3.116.035238)
Supplement: Supplementary file 6 [file 179FigureS6.pdf]

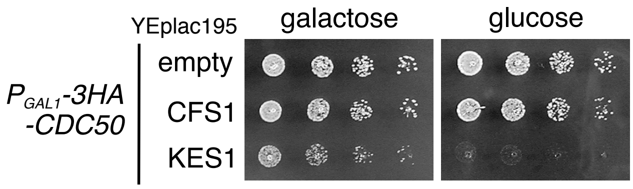

Figure S6 Overexpression of *CFS1* does not affect growth of Cdc50-depleted cells. Five-fold serial dilutions of exponentially growing cultures were spotted onto SGA-Ura (galactose) and SDA-Ura (glucose) plates, followed by incubation at 30°C for 2 days or one day, respectively. The strain used was P_{GAL1}-*3HA-CDC50* (YKT1638), which contains YEplac195 multicopy plasmid harboring the indicated gene.
