# Supplementary material for: Cfs1p, a Novel Membrane Protein in the PQ-Loop Family, Is Involved in Phospholipid Flippase Functions in Yeast
Source: G3 (Bethesda). 2016 Nov 8;7(1):179–92. doi: 10.1534/g3.116.035238 (PMC5217107; doi:10.1534/g3.116.035238)
Supplement: Supplementary file 7 [file 179TableS1.pdf]

Table S1 Strain list

Strain	Genotype	Source or reference
YEF473	*MAT*a/α *his3Δ-200*/*his3Δ-200 leu2Δ-1*/*leu2Δ-1 lys2-82*/ *lys2-82 trp1Δ-63*/*trp1Δ-63 ura3-52*/ *ura3-52*	Bi and Pringle 1996
YKT1066	*MAT*a *his3Δ-200 leu2Δ-1 lys2-82 TRP1 ura3-52*	Zendeh-boodi *et al*. 2013
YKT249	*MAT*a *cdc50Δ::HIS3MX6 trp1Δ-63*	Misu *et al*. 2003
YKT1507	*MAT*a *cdc50Δ::HIS3MX6 TRP1*	Takeda *et al*. 2014
YKT2024	*MAT*a *ymr010w::Tn3-lacZ-LEU2 cdc50Δ::HIS3MX6 trp1Δ-63*	This study
YKT2070	*MAT*a *cfs1Δ::KanMX4 TRP1*	This study
YKT2025	*MAT*a *cfs1Δ::KanMX4 cdc50Δ::HIS3MX6 TRP1*	This study
YKT2035	*MAT*a *kes1Δ::KanMX4 TRP1*	This study
YKT2026	*MAT*a *kes1Δ::KanMX4 cdc50Δ::HIS3MX6 TRP1*	This study
YKT2029	*MAT*a *fun26Δ::KanMX4 TRP1*	This study
YKT2030	*MAT*a *fun26Δ::KanMX4 cdc50Δ::HIS3MX6 TRP1*	This study
YKT2031	*MAT*a *plb3Δ::KanMX4 TRP1*	This study
YKT2032	*MAT*a *plb3Δ::KanMX4 cdc50Δ::HIS3MX6 TRP1*	This study
YKT1636	*MAT*a *drs2Δ::HphMX4 TRP1*	This study
YKT2081	*MAT*a *cfs1Δ::KanMX4 drs2Δ::HphMX4 TRP1*	This study
YKT2082	*MAT*a *kes1Δ::KanMX4 drs2Δ::HphMX4 TRP1*	This study
YKT2039	*MAT*a *rcy1Δ::HphMX4 TRP1*	This study
YKT2083	*MAT*a *cfs1Δ::KanMX4 rcy1Δ::HphMX4 TRP1*	This study
YKT2084	*MAT*a *kes1Δ::KanMX4 rcy1Δ::HphMX4 TRP1*	This study
YKT1890	*MAT*a *HIS3MX6::*P_{GAL1}*-3HA-CDC50 lem3Δ::TRP1*	This study
YKT2045	*MAT*a *HIS3MX6::*P_{GAL1}*-3HA-CDC50 lem3Δ::TRP1 cfs1Δ::CaURA3*	This study
YKT1120	*MAT*a *HIS3MX6::*P_{GAL1}*-3HA-CDC50 lem3Δ::TRP1 crf1Δ::HphMX4*	Yamagami *et al*. 2015
YKT2046	*MAT*a *HIS3MX6::*P_{GAL1}*-3HA-CDC50 lem3Δ::TRP1 crf1Δ::HphMX4 cfs1Δ::CaURA3*	This study
YKT2018	*MAT*a *TRP1::*P_{GAL1}*-NEO1*	This study
YKT2085	*MAT*a *TRP1::*P_{GAL1}*-NEO1 cfs1Δ::CaURA3*	This study
YKT2086	*MAT*a *TRP1::*P_{GAL1}*-NEO1 KanMX6::*P_{GAL1}*-3HA-CDC50 cfs1Δ::CaURA3*	This study
YKT2087	*MAT*a *TRP1::*P_{GAL1}*-NEO1 rcy1Δ::HphMX4 cfs1Δ::CaURA3*	This study
YKT2037	*MAT*a *cfs1Δ::HphMX4 TRP1*	This study
YKT2049	*MAT*a *cdc50Δ::HIS3MX6 lem3Δ::TRP1 cfs1Δ::KanMX4*	This study
YKT2050	*MAT*a *cdc50Δ::HIS3MX6 lem3Δ::TRP1 crf1Δ::HphMX4 cfs1Δ::KanMX4*	This study
YKT2088	*MAT*a *cdc50Δ::HIS3MX6 lem3Δ::TRP1 crf1Δ::HphMX4 kes1Δ::KanMX4*	This study
YKT2051	*MAT*a *neo1Δ::HIS3MX6 cfs1Δ::HphMX4 TRP1*	This study
YKT1523	*MAT*a *URA3::*P_{TPI1}*-GFP-SNC1 TRP1*	Takeda *et al*. 2014
YKT2055	*MAT*α *URA3::*P_{TPI1}*-GFP-SNC1 cfs1Δ::HIS3MX4 TRP1*	This study

Strain	Genotype	Source or reference
YKT2056	*MAT*a *URA3::P$_{TPI1}$-GFP-SNC1 KanMX6::P$_{GAL1}$-3HA-CDC50 TRP1*	This study
YKT2057	*MAT*α *URA3::P$_{TPI1}$-GFP-SNC1 KanMX6::P$_{GAL1}$-3HA-CDC50 cfs1*Δ*::HIS3MX4 TRP1*	This study
YKT2058	*MAT*a *URA3::P$_{TPI1}$-GFP-SNC1 KanMX6::P$_{GAL1}$-3HA-CDC50 lem3*Δ*::TRP1*	This study
YKT2059	*MAT*α *URA3::P$_{TPI1}$-GFP-SNC1 KanMX6::P$_{GAL1}$-3HA-CDC50 lem3*Δ*::TRP1 cfs1*Δ*::HIS3MX4*	This study
YKT2060	*MAT*α *URA3::P$_{TPI1}$-GFP-SNC1 KanMX6::P$_{GAL1}$-3HA-CDC50 lem3*Δ*::TRP1 crf1*Δ*::HphMX4*	This study
YKT2061	*MAT*α *URA3::P$_{TPI1}$-GFP-SNC1 KanMX6::P$_{GAL1}$-3HA-CDC50 lem3*Δ*::TRP1 crf1*Δ*::HphMX4 cfs1*Δ*::HIS3MX4*	This study
YKT2062	*MAT*α *URA3::P$_{TPI1}$-GFP-SNC1 KanMX6::P$_{GAL1}$-3HA-NEO1 TRP1*	This study
YKT2063	*MAT*a *URA3::P$_{TPI1}$-GFP-SNC1 KanMX6::P$_{GAL1}$-3HA-NEO1 cfs1*Δ*::HIS3MX4 TRP1*	This study
YKT2093	*MAT*a/α *CFS1-EGFP::KanMX6/CFS1-EGFP::KanMX6 DRS2-mRFP1::TRP1/DRS2-mRFP1::TRP1*	This study
YKT2094	*MAT*a/α *CFS1-EGFP::KanMX6/CFS1-EGFP::KanMX6 NEO1-mRFP1::TRP1/NEO1-mRFP1::TRP1*	This study
YKT2111	*MAT*a/α *CFS1-EGFP::KanMX6/CFS1-EGFP::KanMX6 SEC7-mRFP1::TRP1/SEC7-mRFP1::TRP1*	This study
YKT2020	*MAT*a *ric1*Δ*::TRP1*	This study
YKT2033	*MAT*a *ric1*Δ*::TRP1 cfs1*Δ*::HIS3MX4*	This study
YKT2022	*MAT*a *rgp1*Δ*::TRP1*	This study
YKT2034	*MAT*a *rgp1*Δ*::TRP1 cfs1*Δ*::HIS3MX4*	This study
YKT2095	*MAT*a *URA3::P$_{TPI1}$-GFP-SNC1 ric1*Δ*::TRP1*	This study
YKT2096	*MAT*a *URA3::P$_{TPI1}$-GFP-SNC1 ric1*Δ*::TRP1 cfs1*Δ*::HIS3MX4*	This study
YKT2097	*MAT*a *URA3::P$_{TPI1}$-GFP-SNC1 rgp1*Δ*::TRP1*	This study
YKT2098	*MAT*a *URA3::P$_{TPI1}$-GFP-SNC1 rgp1*Δ*::TRP1 cfs1*Δ*::HIS3MX4*	This study
YKT715	*MAT*a *lem3*Δ*::TRP1*	Saito *et al*. 2004
YKT2099	*MAT*a *lem3*Δ*::TRP1 cfs1*Δ*::KanMX4*	This study
YKT2100	*MAT*a *ENA1-GFP::KanMX6 trp1*Δ*-63*	This study
YKT2101	*MAT*α *ENA1-GFP::KanMX6 cfs1*Δ*::HphMX4 trp1*Δ*-63*	This study
YKT2102	*MAT*α *ENA1-GFP::KanMX6 neo1*Δ*::HIS3MX6 cfs1*Δ*::HphMX4 trp1*Δ*-63*	This study
YKT2074	*MAT*a *sec14-3 TRP1*	This study
YKT2075	*MAT*a *sec14-3 kes1*Δ*::KanMX4 TRP1*	This study
YKT2076	*MAT*a *sec14-3 cfs1*Δ*::HphMX4 TRP1*	This study
YKT1638	*MAT*a *HphMX4::P$_{GAL1}$-3HA-CDC50 trp1*Δ*-63*	This study
YKT2069	*MAT*a *TRP1::P$_{GAL1}$-NEO1 kes1*Δ*::KanMX4*	This study

Strain	Genotype	Source or reference
YKT2104	*MAT*a *URA3::P_{TPI1}-GFP-SNC1 neo1Δ::HIS3MX6 cfs1Δ::HphMX4 TRP*	This study
YKT38	*MAT*a *his3Δ-200 leu2Δ-1 lys2-82 trp1Δ-63 ura3-52*	Mochida *et al*. 2002
YKT2079	*MAT*a *CFS1-EGFP::KanMX4 trp1Δ-63*	This study
YKT2105	*MAT*a *CFS1-EGFP::KanMX4 cdc50Δ::HIS3MX6 trp1Δ-63*	This study
YKT2106	*MAT*a *CFS1-EGFP::KanMX4 vrp1Δ::LEU2 trp1Δ-63*	This study
YKT2064	*MAT*a *cfs1Δ::HphMX4 trp1Δ-63*	This study
BY4719	*MAT*a *HIS3 LEU2 LYS2 MET15 trp1Δ-63 ura3Δ0*	Brachmann *et al*. 1998
BY4741	*MAT*a *his3Δ1 leu2Δ0 LYS2 met15Δ0 TRP1 ura3Δ0*	Brachmann *et al*. 1998
BY4743	*MAT*a/α *his3Δ1/his3Δ1 leu2Δ0/leu2Δ0 LYS2/lys2Δ0 met15Δ0/MET15 TRP1/TRP1 ura3Δ0/ura3Δ0*	Brachmann *et al*. 1998
KKT3	*MAT*α *his3Δ1 leu2Δ0 lys2Δ0 met15Δ0 TRP1 ura3Δ0* (BY4743-derived)	This study
KKT9	*MAT*α *cdc50Δ::HphMX4* (KKT3-derived)	Kishimoto *et al*. 2003
KKT479	*MAT*a *cfs1Δ::KanMX4* (BY4741-derived)	a gift from C. Boone
KKT480	*MAT*a *cfs1Δ::KanMX4 cdc50Δ::HphMX4*	This study
KKT481	*MAT*a *ers1Δ::KanMX4* (BY4741-derived)	a gift from C. Boone
KKT482	*MAT*a *ers1Δ::KanMX4 cdc50Δ::HphMX4*	This study
KKT483	*MAT*a *ydr090cΔ::KanMX4* (BY4741-derived)	a gift from C. Boone
KKT484	*MAT*a *ydr090cΔ::KanMX4 cdc50Δ::HphMX4*	This study
KKT485	*MAT*a *ypq1Δ::KanMX4* (BY4741-derived)	a gift from C. Boone
KKT486	*MAT*a *ypq1Δ::KanMX4 cdc50Δ::HphMX4*	This study
KKT487	*MAT*a *ypq2Δ::KanMX4* (BY4741-derived)	a gift from C. Boone
KKT488	*MAT*a *ypq2Δ::KanMX4 cdc50Δ::HphMX4*	This study
KKT489	*MAT*a *ypq3Δ::KanMX4* (BY4741-derived)	a gift from C. Boone
KKT490	*MAT*a *ypq3Δ::KanMX4 cdc50Δ::HphMX4*	This study
KKT473	*MAT*a *his3Δ1 leu2Δ0 lys2Δ0 met15Δ0 trp1Δ-63 ura3Δ0*	This study
KKT475	*MAT*a *cfs1Δ::HIS3MX4 trp1Δ-63* (KKT473-derived)	This study
KKT476	*MAT*a *lem3Δ::KanMX4 trp1Δ-63* (KKT473-derived)	This study
KKT477	*MAT*a *lem3Δ::KanMX4 cfs1Δ::HIS3MX4 trp1Δ-63* (KKT473-derived)	This study
KKT61	*MAT*a *his3Δ1 leu2Δ0 lys2Δ0 MET15 TRP1 ura3Δ0*	Nakano *et al*. 2008
KKT478	*MAT*a *cfs1Δ::KanMX4 TRP1* (KKT61-derived)	This study

YKT strains are isogenic derivatives of YEF473.

KKT strains were constructed using S288C-derived strains, BY4719, BY4741, and BY4743.

Only relevant genotypes are described.

References

Bi, E., and J. R. Pringle, 1996 *ZDS1* and *ZDS2*, genes whose products may regulate Cdc42p in *Saccharomyces cerevisiae*. Mol. Cell. Biol. 16: 5264–5275.

Brachmann, C. B., A. Davies, G. J. Cost, E. Caputo, J. Li *et al.*, 1998 Designer deletion strains derived from *Saccharomyces cerevisiae* S288C: a useful set of strains and plasmids for PCR-mediated gene disruption and other applications. Yeast 14: 115–132.

Kishimoto, T., T. Yamamoto, and K. Tanaka, 2005 Defects in structural integrity of ergosterol and the Cdc50p-Drs2p putative phospholipid translocase cause accumulation of endocytic membranes, onto which actin patches are assembled in yeast. Mol. Biol. Cell 16: 5592–5609.

Misu, K., K. Fujimura-Kamada, T. Ueda, A. Nakano, H. Katoh *et al.*, 2003 Cdc50p, a conserved endosomal membrane protein, controls polarized growth in *Saccharomyces cerevisiae*. Mol. Biol. Cell 14: 730–747.

Mochida, J., T. Yamamoto, K. Fujimura-Kamada, and K. Tanaka, 2002 The novel adaptor protein, Mti1p, and Vrp1p, a homolog of Wiskott-Aldrich syndrome protein-interacting protein (WIP), may antagonistically regulate type I myosins in *Saccharomyces cerevisiae.* Genetics 160: 923–934.

Nakano K., T. Yamamoto, T. Kishimoto, T. Noji, and K. Tanaka, 2008 Protein kinases Fpk1p and Fpk2p are novel regulators of phospholipid asymmetry. Mol Biol Cell. 19: 1783–1797.

Saito, K., K. Fujimura-Kamada, N. Furuta, U. Kato, M. Umeda *et al.*, 2004 Cdc50p, a protein required for polarized growth, associates with the Drs2p P-Type ATPase implicated in phospholipid translocation in *Saccharomyces cerevisiae*. Mol. Biol. Cell 15: 3418–3432.

Takeda, M., K. Yamagami, and K. Tanaka, 2014 Role of phosphatidylserine in phospholipid flippase-mediated vesicle transport in *Saccharomyces cerevisiae*. Eukaryot. Cell 13: 363–375.

Yamagami, K., T. Yamamoto, S. Sakai, T. Mioka, T. Sano *et al.*, 2015 Inositol depletion restores vesicle transport in yeast phospholipid flippase mutants. PLoS One 10: e0120108.

Zendeh-boodi, Z., T. Yamamoto, H. Sakane, and K. Tanaka, 2013 Identification of a second amphipathic lipid-packing sensor-like motif that contributes to Gcs1p function in the early endosome-to-TGN pathway. J. Biochem. 153: 573–587
